# Chemotherapy-induced adenosine A2B receptor expression mediates epigenetic regulation of pluripotency factors and promotes breast cancer stemness

**DOI:** 10.7150/thno.70581

**Published:** 2022-02-28

**Authors:** Jie Lan, Guangyao Wei, Jia Liu, Fan Yang, Rong Sun, Haiquan Lu

**Affiliations:** 1 Department of Radiation Oncology, Department of Thoracic Oncology, Cancer Center and State Key Laboratory of Biotherapy, West China Hospital, Sichuan University, Chengdu, Sichuan 610041, China; 2 Advanced Medical Research Institute and Key Laboratory for Experimental Teratology of the Ministry of Education, Cheeloo College of Medicine, Shandong University, Jinan, Shandong 250012, China; 3 The Second Hospital, Cheeloo College of Medicine, Shandong University, Jinan, Shandong 250033, China

**Keywords:** chemotherapy, adenosine receptor, breast cancer stem cell, pluripotency factors, epigenetic regulation

## Abstract

**Rationale:** Triple-negative breast cancer (TNBC) is characterized by its unique molecular profile, aggressive nature and lack of targeted therapy. Chemotherapy induces expression of pluripotency factors and mediates an active induction of breast cancer stem cells (BCSCs) in TNBC, which potentiates the risk of tumor recurrence and metastasis and increases patient mortality. Adenosine receptor 2B (A2BR) expression and activation of its downstream signaling pathway has been implied to promote breast cancer metastasis. This study is to investigate the role of A2BR in the regulation of chemotherapy-induced BCSC enrichment.

**Methods:** We generated shRNA-mediated A2BR knockdown subclones in TNBC cell lines and evaluated the effect on the BCSC phenotype by Aldefluor and mammosphere assays in vitro. We performed chromatin immunoprecipitation (ChIP) assay to investigate recruitment of transcription factor FOXO3 and histone modification enzymes KDM6A and p300 to the regulatory regions of pluripotency factors, as well as levels of histone modification marks H3K27ac and H3K27me3 on these regions. We employed both xenograft model and genetically engineered, autochthonous breast cancer model to evaluate the effect of A2BR on chemotherapy-induced BCSC enrichment in vivo.

**Results:** We demonstrated that chemotherapy increased protein level of A2BR, which contributed to chemotherapy-induced pluripotency factor expression and BCSC enrichment in TNBC. A2BR mediated activation of p38 MAPK and nuclear translocation of chromatin remodeling factor SMARCD3, which interacted and recruited histone demethylase KDM6A and histone acetyltransferase p300 specifically to the pluripotency factor genes *NANOG*, *SOX2* and *KLF4*. Recruitment of KDM6A and p300 decreased histone H3K27me3 and increases H3K27ac marks, and increased transcription factor FOXO3 binding to *NANOG*, *SOX2* and *KLF4* genes, leading to transcriptional activation of these genes and BCSC specification. Genetic or pharmacological inhibition of A2BR blocked chemotherapy-mediated epigenetic activation of pluripotency factor genes and BCSC enrichment in vitro and in vivo, and delayed tumor recurrence after chemotherapy was discontinued.

**Conclusion:** Chemotherapy-induced A2BR expression mediates epigenetic activation of pluripotency factors and promotes breast cancer stemness. Targeting A2BR in combination with chemotherapy may block BCSC enrichment and improve outcome in TNBC.

## Introduction

Triple-negative breast cancer (TNBC) is a subtype of breast cancer that lacks expression of estrogen receptor (ER), progesterone receptor (PR) and human epidermal growth factor receptor 2 (HER2) 1. TNBC is highly aggressive: Approximately 46% of TNBC patients develops distant metastasis, which is the major cause of patient mortality, and the median survival of patients with metastatic TNBCs is only 13.3 months 2. TNBC has a poor prognosis due in part to lack of targeted therapy 3. As the mainstay of treatment for TNBC, cytotoxic chemotherapy reduces tumor bulk initially, but the majority of patients have residual disease or relapse 4. Therefore, a better understanding of the mechanism underlying chemotherapy resistance is urgently needed to improve outcome of TNBC therapy.

Breast cancer stem cells (BCSCs) are a small and dynamic subpopulation of breast cancer cells that play a critical role in cancer metastasis 5. BCSCs have infinite proliferative potential and tumor-initiating properties, and are resistant to chemotherapy treatment 6,7. Our previous studies have demonstrated an average of 4-fold increase in BCSC population after treatment with different chemotherapy drugs at the concentration of the drug that killed 50% of the cancer cells (IC_50_) 8-10, suggesting that differential survival to chemotherapy between BCSCs and non-stem cells alone cannot account for the observed difference in the percentage of BCSCs before and after chemotherapy. Explanation to this paradox is that chemotherapy induces an active conversion from non-stem cells to BCSCs.

A major mechanism underlying chemotherapy-induced BCSC enrichment is the increased expression of pluripotency factors NANOG, SOX2, OCT4, and KLF4 in response to chemotherapy 8-11. Pluripotency factors are master regulators of self-renewal and pluripotency in embryonic stem cells (ESCs) 12 and are also required for the maintenance and specification of BCSCs 13-15. Regulation and roles of pluripotency factors are well elucidated in ESCs, where they coordinately activate the expression of pluripotency-associated factors and form a feed-forward loop to regulate the expression of each other and that of their own 16. However, in cancer stem cells, the molecular mechanisms underlying regulation of pluripotency factor expression remain elusive.

The expression of pluripotency factors in cancer stem cells has been reported to be regulated at transcriptional level by different transcription factors in addition to themselves, including STAT3, HIF-1, GLI1, AR, and FOXO3 8,17. Epigenetic regulation of chromatin structure plays a critical role in the activation or repression of pluripotency factor gene transcription by controlling transcription factor accessibility to genomic DNA 18. DNA accessibility is regulated through dynamic modification of chromatin architecture, i.e., chromatin remodeling, by two classes of enzyme complexes: ATP-dependent chromatin remodeling complexes that reposition nucleosomes along DNA and evict histones from DNA, and histone modifying enzymes that covalently modify histone tails 19,20. Our recent work has demonstrated that histone demethylase KDM6A/UTX plays an important role in the regulation of pluripotency factor genes transcription in response to chemotherapy in breast cancer 21. How ATP-dependent chromatin remodeling complexes are involved in this process is largely unknown.

Accumulation of extracellular adenosine, sequentially converted from extracellular ATP by CD39 and CD73 during tumor growth 22, has been implicated in chemoresistance and metastasis 23. Chemotherapy treatment increases extracellular adenosine levels through induction of CD39 and CD73 expression in breast cancer 24. Adenosine elicits its function by binding to its four cognate receptors (A1R, A2AR, A2BR, and A3R) that belong to the G-protein-coupled receptor (GPCR) superfamily, and the biological consequences of its binding with different receptors are diverse 25. Among these adenosine receptors, A2BR (encoded by *ADORA2B* gene) expression and activation of its downstream signaling pathway has been shown to promote breast cancer metastasis 26,27, and genetic inhibition of A2BR blocks lung metastasis of TNBC in a xenograft mouse model 27. In the present study, we found that chemotherapy induces A2BR expression that leads to activation of p38 MAPK, which mediates nuclear translocation of chromatin remodeling factor SMARCD3 and recruitment of demethylase KDM6A and acetyltransferase p300 to pluripotency factor *NANOG*, *SOX2*, and *KLF4* genes. Reciprocal modification of H3K27me3 and H3K27ac marks increases transcription factor FOXO3 binding and transcriptional activation of pluripotency factor genes, leading to BCSC specification.

## Results

### Chemotherapy-induced A2BR expression promotes pluripotency factor expression and the BCSC phenotype

**Figure 1:**
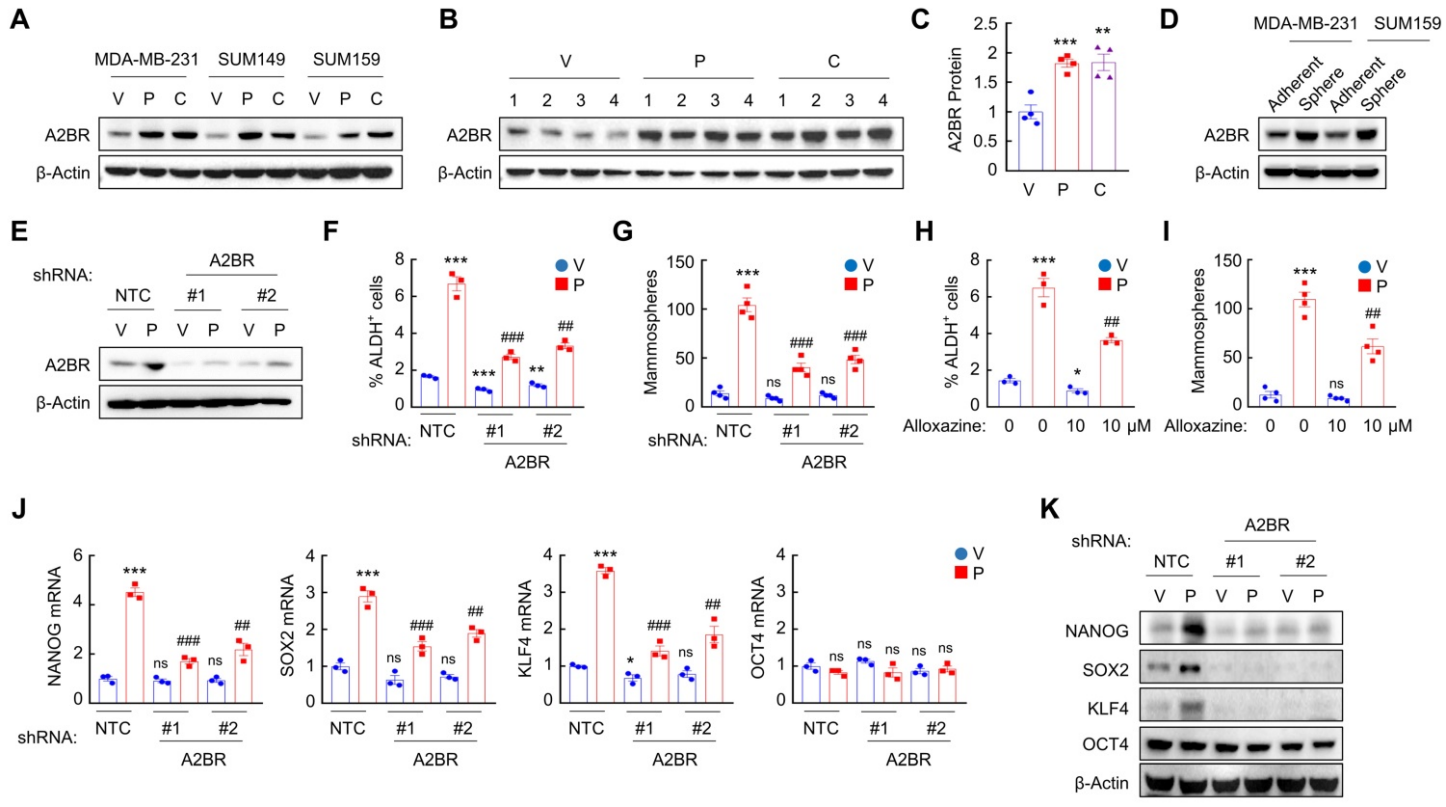
** Chemotherapy-induced A2BR expression promotes pluripotency factor expression and the BCSC phenotype. A,** Breast cancer cell lines were treated with vehicle (V), paclitaxel (P), or carboplatin (C) for 72 h at IC_50_, and immunoblot assay was performed to analyze A2BR protein expression. **B** and** C,** MDA-MB-231 cells were implanted into the mammary fat pad (MFP) of female SCID mice. When tumor volume reached 200 mm^3^ (day 0), mice were randomly assigned to treatment with V, P (10 mg/kg on days 0, 5, 10), or C (20 mg/kg on days 0, 5, 10). Tumors were harvested on day 13 for immunoblot assay. Densitometric analysis of immunoblot (B) was performed and result (C) is presented as mean ± SEM (n = 4); **p < 0.01, ***p < 0.001 vs. V. **D,** MDA-MB-231 and SUM159 cells were cultured on standard polystyrene tissue culture plates (Adherent) or ultra-low adherence plates (Sphere) for 7 days and harvested for A2BR protein expression. **E**-**G,** MDA-MB-231 subclones stably transfected with vector encoding non-targeting control shRNA (NTC) or either of two different shRNAs targeting A2BR (#1 and #2) were treated with vehicle (V) or 10 nM paclitaxel (P) for 72 h. Expression of A2BR protein (E), the percentage of ALDH^+^ cells (F; mean ± SEM; n = 3) and the number of mammospheres formed per 1,000 cells seeded (G; mean ± SEM; n = 4) were determined; **p < 0.01, ***p < 0.001 vs. NTC-V; ^##^p < 0.01, ^###^p < 0.001 vs. NTC-P; ns, not significant. **H** and** I,** MDA-MB-231 cells were treated with 10 nM paclitaxel, 10 μM alloxazine, or both for 72 h. The percentage of ALDH^+^ cells (H; mean ± SEM; n = 3) and the number of mammospheres formed per 1,000 cells seeded (I; mean ± SEM; n = 4) were determined; *p < 0.05, ***p < 0.001 vs. V; ^##^p < 0.01 vs. P; ns, not significant. **J** and** K,** MDA-MB-231 NTC or A2BR knockdown subclones were treated with V or 10 nM P for 72 h, and RT-qPCR (J; mean ± SEM; n = 3) and immunoblot (K) assays were performed. In (J), *p < 0.05, ***p < 0.001 vs. NTC-V; ^##^p < 0.01, ^###^p < 0.001 vs. NTC-P; ns, not significant.

To explore the effect of chemotherapy on A2BR expression, we treated TNBC cell lines MDA-MB-231 (invasive ductal carcinoma cell line with *BRAF*, *CDKN2A*, *KRAS*, and *TP53* mutation), SUM149 (inflammatory ductal carcinoma cell line with *BRCA1* mutation), and SUM159 (anaplastic carcinoma cell line with *PIK3CA* and *TP53* mutation) with Food and Drug Administration (FDA)-approved chemotherapy drugs paclitaxel and carboplatin for 72 h at the IC_50_ of the drug, and found that each of these chemotherapeutic drugs increased A2BR protein levels in all these cell lines (Figure 1A). We implanted MDA-MB-231 cells into the mammary fat pad (MFP) of female severe combined immunodeficiency (SCID) mice and treated the mice with 10 mg/kg paclitaxel or 20 mg/kg carboplatin every 5 days, and found that both chemotherapy drugs increased A2BR protein levels in vivo (Figure 1B-C).

A2BR expression has been reported to promote tumor metastasis in breast cancer 26,27. Because BCSCs are the subpopulation of cancer cells that are capable of forming a clinically relevant metastatic tumor 6, we investigated the role of A2BR in the regulation of the BCSC phenotype. We cultured MDA-MB-231 and SUM159 cells as mammospheres, which enriched BCSC population, and found that A2BR protein levels were significantly higher in nonadherent mammosphere cultures as compared to monolayer cultures in both cell lines (Figure 1D), suggesting a correlation between A2BR expression and the BCSC phenotype.

To investigate the role of A2BR in chemotherapy-induced BCSC enrichment, we generated shRNA-mediated non-targeting control (NTC) or A2BR knockdown subclones of MDA-MB-231 (Figure 1E) and SUM159 (Figure S1A) cells, treated them with paclitaxel for 72 h, and performed Aldefluor and mammosphere assays to measure BCSC population. Paclitaxel treatment of the NTC subclones significantly increased the percentage of cells with aldehyde dehydrogenase activity (ALDH^+^) (Figure 1F and Figure S1B), and increased the number of mammosphere-forming cells (Figure 1G and Figure S1C). A2BR knockdown abrogated paclitaxel-induced enrichment of ALDH^+^ and mammosphere-forming cells in both cell lines (Figure 1F-G; Figure S1B-C). Pharmacological inhibition of A2BR by coadministration of specific A2BR antagonist alloxazine 28 also markedly attenuated paclitaxel-induced enrichment of ALDH^+^ and mammosphere-forming cells in both cell lines (Figure 1H-I; Figure S1D-E), indicating that A2BR expression and activity are required for chemotherapy-induced enrichment of BCSCs. A2BR knockdown also blocked paclitaxel-induced expression of pluripotency factors NANOG, SOX2, and KLF4 (but not OCT4, whose expression is not induced by paclitaxel treatment), which are required for the maintenance and specification of BCSCs, at both mRNA (Figure 1J and Figure S1F) and protein (Figure 1K) levels in MDA-MB-231 and SUM159 cell lines. Taken together, these data demonstrate that chemotherapy drugs induce A2BR protein expression, which is required for chemotherapy-induced pluripotency factor expression and BCSC enrichment.

### Inhibition of A2BR blocks paclitaxel-induced BCSC enrichment and delays tumor recurrence in vivo

**Figure 2:**
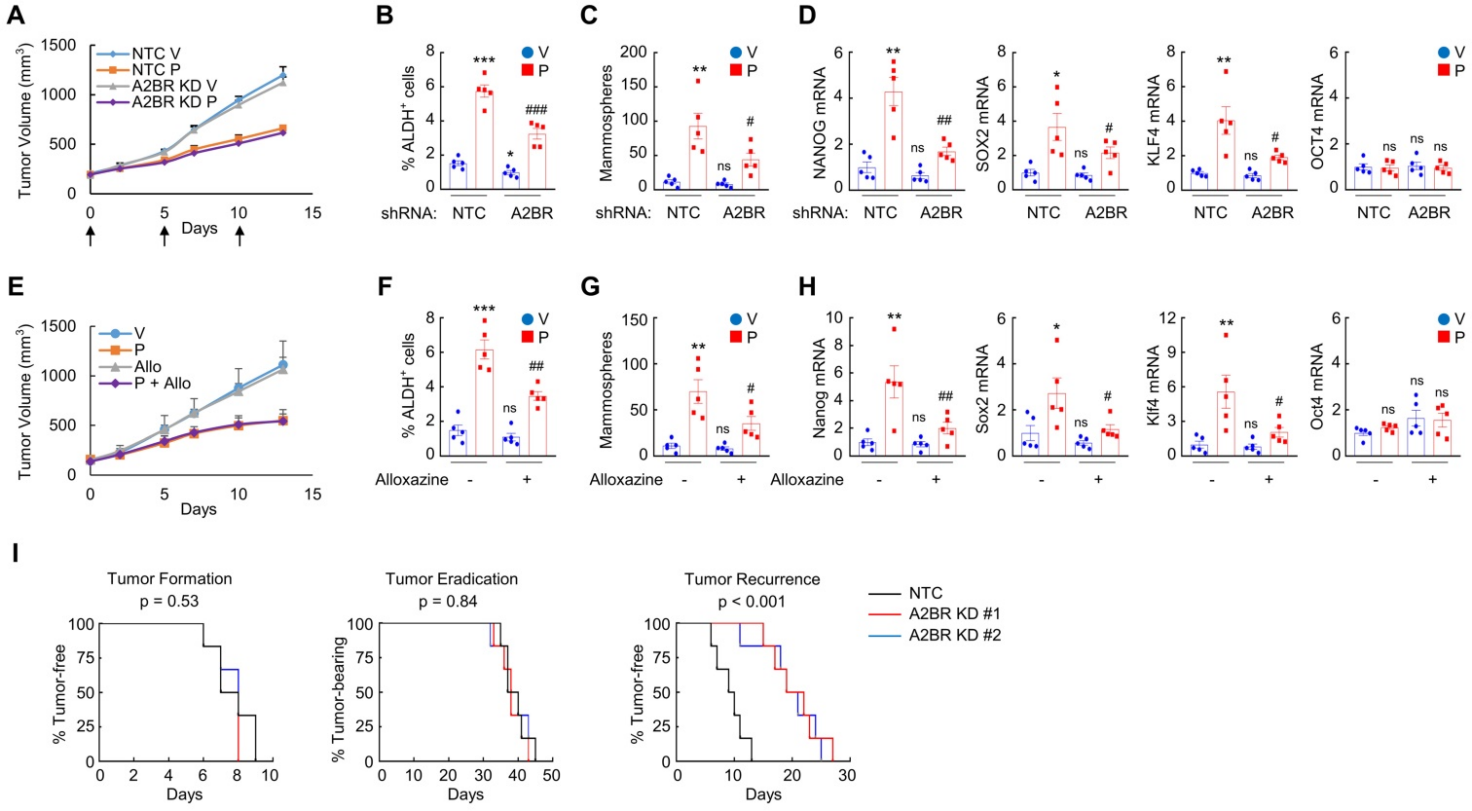
** Inhibition of A2BR blocks paclitaxel-induced BCSC enrichment and delays tumor recurrence in vivo. A**-**D,** 2 x 10^6^ MDA-MB-231 NTC or A2BR knockdown (A2BR KD) subclone cells were implanted into the MFP of SCID mice. When tumor volume reached 200 mm^3^ (day 0), mice were grouped randomly and treated with vehicle (V) or paclitaxel (P; 10 mg/kg, days 0, 5, and 10), and tumor volumes were measured every 2-3 days (A). Tumors were harvested on day 13 for ALDH (B), mammosphere (C), and RT-qPCR (D) assays. Data are shown as mean ± SEM (n = 5); *p < 0.05, **p < 0.01, ***p < 0.001 vs. NTC-V; ^#^p < 0.05, ^##^p < 0.01 vs. NTC-P; ns, not significant. **E**-**H,** MMTV-PyMT transgenic mice were treated with vehicle (V), paclitaxel (P; 5 mg/kg, days 0, 5, and 10), alloxazine (Allo; 10 mg/kg, days 0-13), or P + Allo, when tumors reached a cumulative volume of 150 mm^3^. Tumor volumes were measured every 2-3 days (E). Tumors were harvested on day 13 for ALDH (F), mammosphere (G), and RT-qPCR (H) assays. Data are shown as mean ± SEM (n = 5); *p < 0.05, **p < 0.01, ***p < 0.001 vs. V; ^#^p < 0.05, ^##^p < 0.01 vs. P; ns, not significant. **I,** 2 x 10^6^ MDA-MB-231 NTC or A2BR KD subclone cells were implanted into the MFP of SCID mice. When tumors were palpable, mice were treated with 10 nM paclitaxel every 5 days until tumors were no longer palpable. Kaplan-Meier survival curves of tumor-free (left), tumor-bearing (center), and recurrence-free (right) were plotted and p values of log-rank tests were shown (n = 6).

Next, we investigated the role of A2BR in the regulation of chemotherapy-induced BCSC enrichment in vivo. We injected 2 × 10^6^ MDA-MB-231 NTC or A2BR knockdown subclone cells into the MFP of SCID mice, and when the tumor volume reached 200 mm^3^, the mice were treated with 10 mg/kg of paclitaxel by intraperitoneal injection every 5 days for three doses. Tumors were harvested for ALDH, mammosphere, and qPCR assays 3 days after the last dose. A2BR knockdown did not affect tumor growth rate or sensitivity to paclitaxel (Figure 2A), but attenuated paclitaxel-mediated increases of the percentage of ALDH^+^ cells (Figure 2B), the number of mammosphere-forming cells (Figure 2C), and the mRNA expression of pluripotency factors NANOG, SOX2, and KLF4 (Figure 2D).

To explore the effect of pharmacological inhibition of A2BR on paclitaxel-induced BCSC enrichment in vivo, we employed a genetically engineered, autochthonous breast cancer model and treated MMTV-PyMT transgenic mice with 5 mg/kg paclitaxel every 5 days, alone or in combination with 10 mg/kg alloxazine daily. Paclitaxel treatment also increased the percentage of ALDH^+^ cells (Figure 2F), the number of mammosphere-forming cells (Figure 2G), and Nanog, Sox2, and Klf4 mRNA levels (Figure 2H) in MMTV-PyMT transgenic mice. Coadministration of alloxazine markedly blocked paclitaxel-induced BCSC enrichment and pluripotency factor expression (Figure 2F-H), without affecting growth rate of primary tumors (Figure 2E).

We also implanted 2 × 10^6^ MDA-MB-231 NTC or A2BR knockdown subclone cells into the MFP, treated the mice with 10 mg/kg of paclitaxel every 5 days when tumors became palpable, terminated treatment when tumors were no longer palpable, and monitored the mice for tumor recurrence. A2BR knockdown did not alter time for tumor formation (Figure 2I, left panel) or tumor eradication (Figure 2I, middle panel), but markedly increased time for tumor recurrence (Figure 2I, right panel), indicating that A2BR knockdown inhibits tumor relapse, a process majorly attributed to existence of BCSCs. Taken together, these data demonstrate the critical role of A2BR in paclitaxel-induced BCSC enrichment and tumor recurrence in vivo.

### A2BR promotes FOXO3 binding on pluripotency factor genes through reciprocal regulation of H3K27me3 and H3K27ac chromatin marks

**Figure 3:**
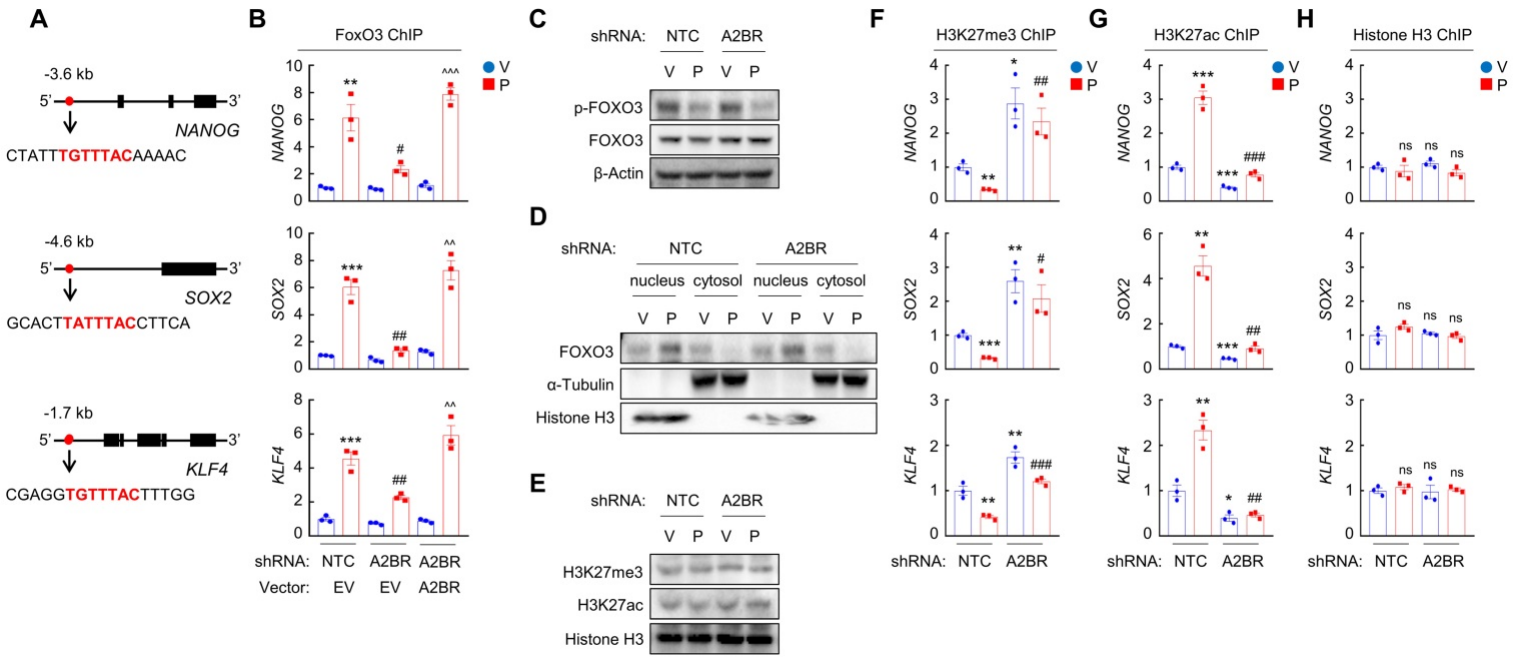
** Chemotherapy-induced A2BR expression promotes FOXO3 binding on pluripotency factor genes through decreased H3K27me3 and increased H3K27ac chromatin marks. A** and **B,** MDA-MB-231 NTC or A2BR knockdown subclones were transfected with pLX304 (empty vector, EV) or pLX304 encoding A2BR. Cells were treated with vehicle (V) or 10 nM paclitaxel (P) for 72 h and chromatin immunoprecipitation (ChIP) was performed with antibody against FOXO3. Primers flanking the FOXO3 binding site in the *NANOG*, *SOX2* and* KLF4* gene (A) were used for qPCR (B; mean ± SEM; n = 3); **p < 0.01, ***p < 0.001 vs. NTC/EV-V; ^#^p < 0.05, ^##^p < 0.01 vs. NTC/EV-P; ^^^^p < 0.01, ^^^^^p < 0.0001 vs. A2BR shRNA/EV-P; ns, not significant. **C** and **E,** MDA-MB-231 NTC or A2BR knockdown subclones were treated with V or P for 72 h and immunoblot assays were performed. **D,** MDA-MB-231 NTC or A2BR knockdown subclones were treated with V or P for 72 h. Cytosolic and nuclear lysates were prepared, and immunoblot assays were performed. **F-H,** MDA-MB-231 NTC or A2BR knockdown subclones were treated with V or 10 nM P for 72 h. ChIP was performed using antibodies against H3K27me3 (F), H3K27ac (G), or Histone H3 (H) followed by qPCR with primers flanking FOXO3 binding sites in the *NANOG*, *SOX2* and* KLF4* gene (mean ± SEM; n = 3); *p < 0.05, **p < 0.01, ***p < 0.001 vs. NTC-V; ^#^p < 0.05, ^##^p < 0.01, ^###^p < 0.001 vs. NTC-P; ns, not significant.

We next investigated the mechanism by which A2BR regulates pluripotency factor expression, which is critical for the specification of the BCSC phenotype. Previously we have reported that transcription factor FOXO3 activates expression of pluripotency factors in response to chemotherapy in TNBC 8. To explore the involvement of FOXO3 in A2BR-induced pluripotency factor expression in response to chemotherapy, we searched genomic DNA sequences for matches to the consensus FOXO3 binding-site sequence 5'-T(A/G)TTTAC-3' 29 and performed chromatin immunoprecipitation (ChIP) to evaluate FOXO3 binding on pluripotency factor genes in NTC and A2BR knockdown subclones of MDA-MB-231 and SUM159 cells. Paclitaxel treatment induced binding of FOXO3 to pluripotency factor *NANOG*, *SOX2*, and *KLF4* genes (Figure 3A-B; Figure S2A). A2BR knockdown abrogated paclitaxel-induced FOXO3 binding on pluripotency factor genes, which was rescued by transfection with an shRNA-resistant A2BR expression vector (Figure 3B and Figure S2A). These data indicate that A2BR promotes NANOG, SOX2, and KLF4 expression through regulation of transcription factor FOXO3 binding on the regulatory region of these genes.

We then investigated how A2BR regulates FOXO3 binding on pluripotency factor genes. Paclitaxel treatment decreased FOXO3 phosphorylation at S294 (Figure 3C) and promoted its nuclear translocation, without affecting total FOXO3 expression (Figure 3D). However, A2BR knockdown did not affect paclitaxel-mediated FOXO3 dephosphorylation and nuclear localization (Figure 3C-D), suggesting that A2BR regulates FOXO3 transcriptional activity independent of its subcellular localization. Next, we explored whether chromatin accessibility at the FOXO3 binding site of *NANOG*, *SOX2* and *KLF4* genes is regulated by A2BR. We treated NTC or A2BR knockdown subclones of MDA-MB-231 and SUM159 with paclitaxel and performed ChIP with antibodies against H3K27me3, a marker of epigenetically repressed genes, and against H3K27ac, a marker of epigenetically activated genes, followed by qPCR with primers flanking FOXO3 binding site of *NANOG*, *SOX2* and *KLF4* genes. Paclitaxel treatment significantly decreased H3K27me3 and increased H3K27ac marks at the FOXO3 binding sites of the *NANOG*, *SOX2*, and *KLF4* genes; Conversely, A2BR knockdown increased H3K27me3 and decreased H3K27ac marks at the FOXO3 binding sites of these genes (Figure 3F-G; Figure S2B-C). Total histone H3 occupancy at these sites were not affected by either paclitaxel treatment or A2BR knockdown (Figure 3H and Figure S2D). Paclitaxel treatment or A2BR knockdown only affected H3K27me3 and H3K27ac modification specifically at the FOXO3 binding sites of *NANOG*, *SOX2* and *KLF4* genes, but did not affect global H3K27me3 and H3K27ac levels (Figure 3E). These data indicate that A2BR mediates decreased H3K27me3 and increased H3K27ac modification at specific FOXO3 binding sites of *NANOG*, *SOX2* and *KLF4* genes and promotes chromatin accessibility and FOXO3 binding to these regions in response to chemotherapy.

**Figure 4:**
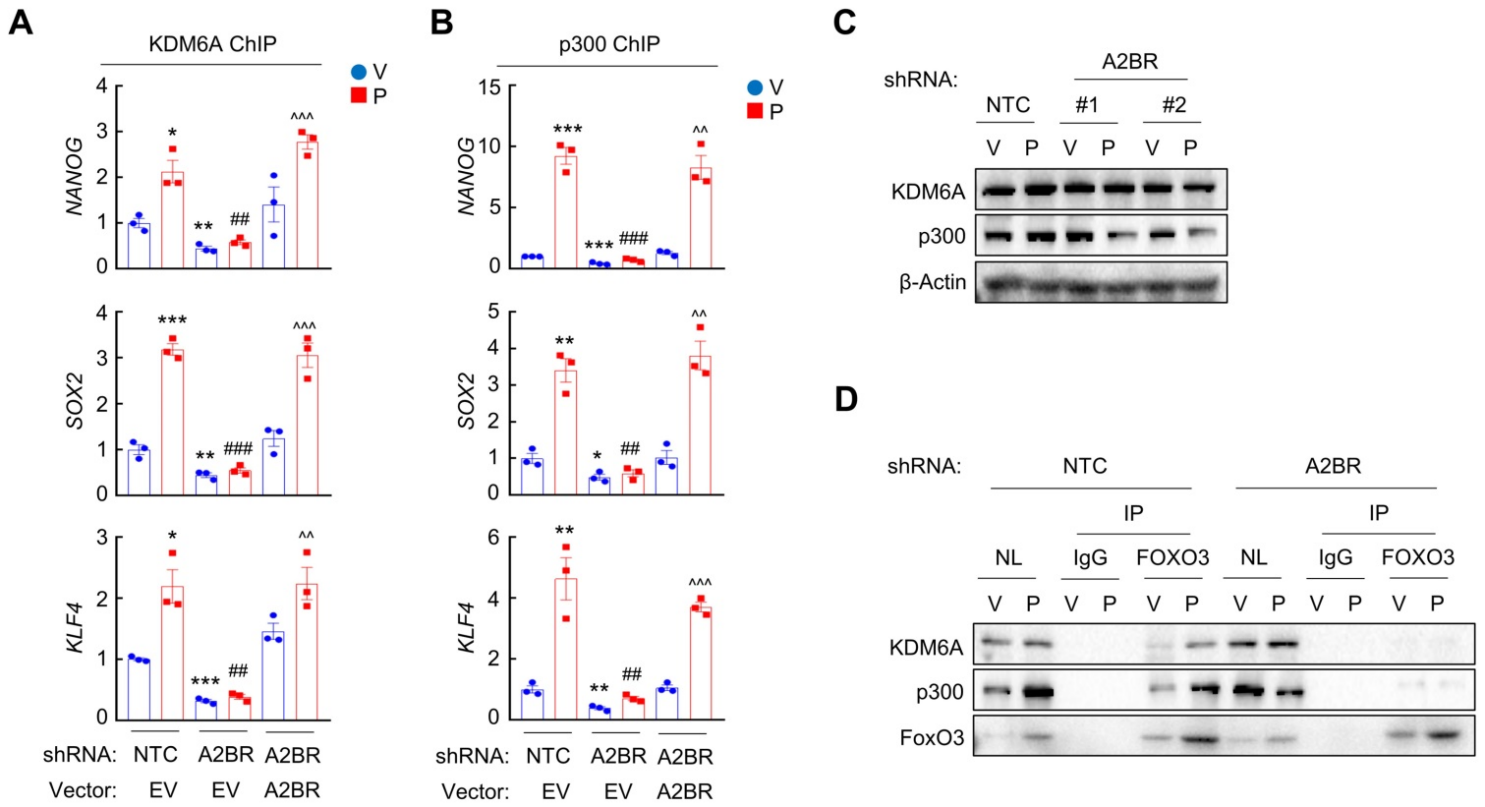
** A2BR decreases H3K27me3 and increases H3K27ac marks through recruitment of KDM6A and p300 at FOXO3 binding sites of pluripotency factor genes. A** and **B,** MDA-MB-231 NTC or A2BR knockdown subclones were transfected with pLX304 (empty vector, EV) or pLX304 encoding A2BR. Cells were treated with vehicle (V) or 10 nM paclitaxel (P) for 72 h. ChIP was performed using antibodies against KDM6A (A) or p300 (B) followed by qPCR with primers flanking FOXO3 binding sites in the *NANOG*, *SOX2* and* KLF4* gene (mean ± SEM; n = 3); *p < 0.05, **p < 0.01, ***p < 0.001 vs. NTC-V; ^##^p < 0.01, ^###^p < 0.001 vs. NTC-P; ^^^^p < 0.01, ^^^^^p < 0.0001 vs. A2BR shRNA/EV-P; ns, not significant. **C,** MDA-MB-231 NTC or A2BR knockdown subclones were treated with V or P for 72 h and immunoblot assays were performed. **D,** MDA-MB-231 NTC or A2BR knockdown subclones were treated with V or P for 72 h and nuclear lysates were prepared. Immunoprecipitation (IP) was performed using FOXO3 antibody or control IgG followed by immunoblot assays. NL, nuclear protein lysate.

Next, we investigated the occupancy of enzymes that regulates H3K27me3 and H3K27ac at the FOXO3 binding sites of *NANOG*, *SOX2* and *KLF4* genes. Paclitaxel treatment increased recruitment of KDM6A (Figure 4A and Figure S3A), the histone demethylase that decreases H3K27me3 marks 30, and increased recruitment of p300 (Figure 4B and Figure S3B), the histone acetyltransferase that increases H3K27ac marks 31, at the FOXO3 binding sites of these genes, without affecting global KDM6A or p300 expression (Figure 4C). Paclitaxel-induced KDM6A and p300 recruitment to FOXO3 binding sites of pluripotency factor genes was abolished by A2BR knockdown and rescued by transfection with an shRNA-resistant A2BR expression vector (Figure 4A-B; Figure S3A-B). Recruitment of KDM6A and p300 to FOXO3 binding sites was further confirmed by co-IP with FOXO3 antibody using nuclear protein lysates from MDA-MB-231 cells. Paclitaxel treatment increased FOXO3 interaction with KDM6A and p300, which was completely abrogated in A2BR knockdown subclone (Figure 4D). These data indicate that A2BR promotes KDM6A and p300 recruitment to the FOXO3 binding sites of *NANOG*, *SOX2* and *KLF4* genes, leading to reciprocal modification of H3K27me3 and H3K27ac marks.

We further confirmed the regulatory role of A2BR on histone H3 modification at specific FOXO3 binding sites of *NANOG*, *SOX2* and *KLF4* genes in vivo. Mice implanted with MDA-MB-231 NTC or A2BR knockdown subclones were treated with 10 mg/kg paclitaxel every 5 days for three doses and tumor samples were collected for ChIP followed by qPCR with primers flanking FOXO3 binding site of *NANOG*, *SOX2* and *KLF4* genes. Paclitaxel treatment increased FOXO3, KDM6A, and p300 binding to these genes, and these effects were abrogated by A2BR knockdown (Figure S4A, E-F). Paclitaxel decreased H3K27me3 and increased H3K27ac marks, whereas A2BR knockdown increased H3K27me3 and decreased H3K27ac marks at FOXO3 binding site of these genes (Figure S4B-C). Total histone H3 occupancy was not altered by either paclitaxel treatment or A2BR knockdown (Figure S4D). Taken together, these data demonstrate that A2BR promotes KDM6A and p300 recruitment to FOXO3 binding sites of *NANOG*, *SOX2* and *KLF4* genes that decreases H3K27me3 and increases H3K27ac marks at these sites, leading to increased chromatin accessibility, transcription factor FOXO3 binding, and finally activation of pluripotency factor genes.

### A2BR promotes FOXO3 binding and expression of pluripotency factor genes through activation of p38 MAPK

**Figure 5:**
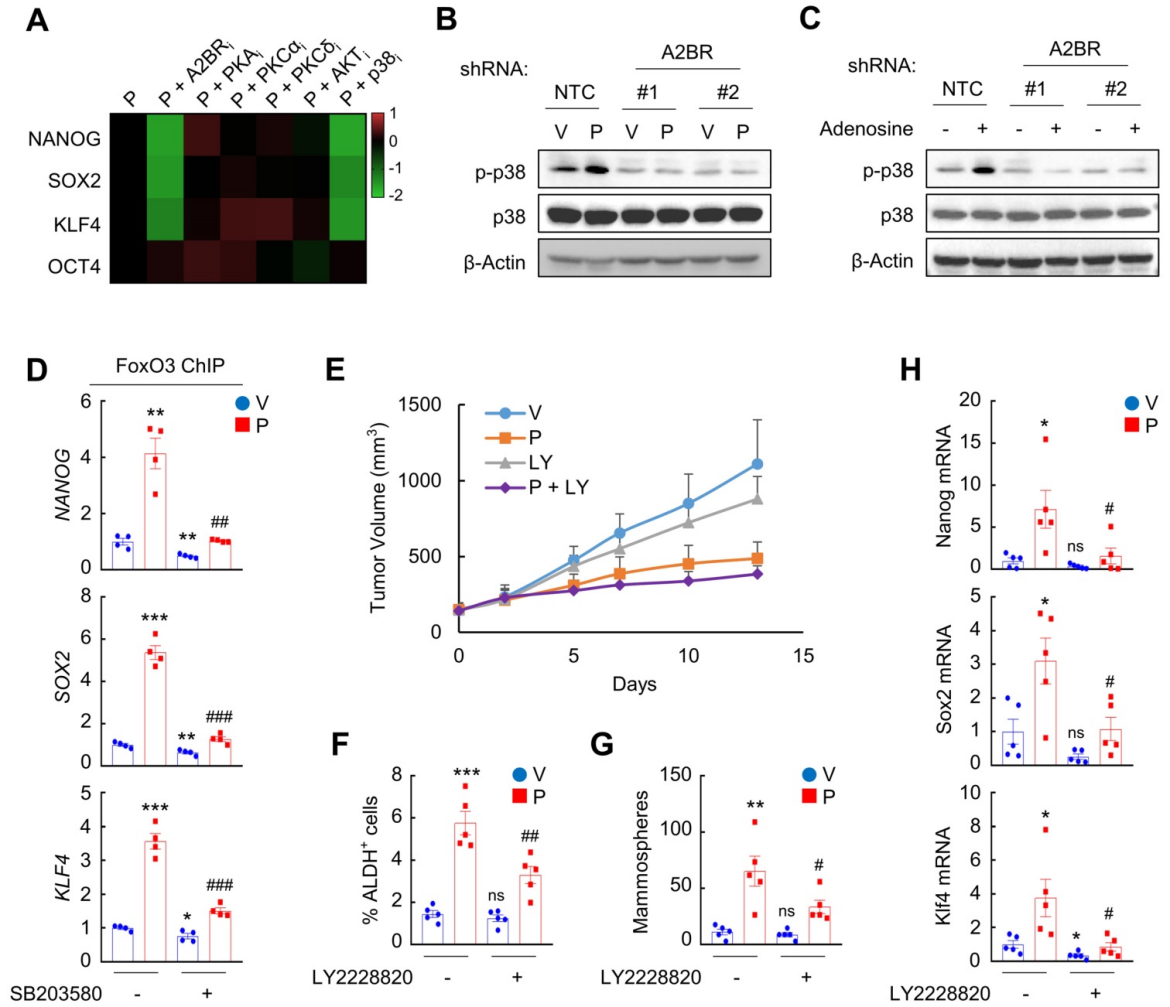
** A2BR promotes FOXO3 binding and expression of pluripotency factor genes through activation of p38 MAPK. A,** MDA-MB-231 cells were treated with 10 nM paclitaxel (P), alone or in combination with 10 μM alloxazine (A2BR_i_), 2.5 μM H-89 (PKA_i_), 1 μM Gö6983 (PKCα_i_), 1 μM rottlerin (PKCδ_i_), 1 μM MK-2206 (AKT_i_), or 5 μM SB203580 (p38_i_), for 72 h. RT-qPCR assays were performed and log_2_ fold change of mRNA expression of pluripotency factors (vs. P) is presented as a heat map. **B,** MDA-MB-231 NTC or A2BR knockdown subclones were treated with vehicle (V) or 10 nM paclitaxel (P) for 72 h and immunoblot assays were performed. **C,** MDA-MB-231 NTC or A2BR knockdown subclones were treated without (-) or with (+) 5 μM adenosine for 72 h and immunoblot assays were performed. **D,** MDA-MB-231 cells were treated with 10 nM paclitaxel, 5 μM SB203580, or both for 72 h. ChIP was performed using FOXO3 antibody followed by qPCR with primers flanking FOXO3 binding sites in the *NANOG*, *SOX2* and* KLF4* gene (mean ± SEM; n = 4); *p < 0.05, **p < 0.01, ***p < 0.001 vs. V; ^##^p < 0.01, ^###^p < 0.001 vs. P. **E**-**H,** MMTV-PyMT transgenic mice were treated with V, P (5 mg/kg, days 0, 5, and 10), LY2228820 (LY; 10 mg/kg, days 0-13), or P + LY, when tumors reached a cumulative volume of 150 mm^3^. Tumor volumes were measured every 2-3 days (E). Tumors were harvested on day 13 for ALDH (F), mammosphere (G), and RT-qPCR (H) assays. Data are shown as mean ± SEM (n = 5); *p < 0.05, **p < 0.01, ***p < 0.001 vs. V; ^#^p < 0.05, ^##^p < 0.01 vs. P; ns, not significant.

We next delineated A2BR downstream signaling pathway that regulates epigenetic regulation of pluripotency factor genes. We treated MDA-MB-231 and SUM159 cells with paclitaxel in combination with A2BR inhibitor alloxazine, or with inhibitors of common A2BR downstream protein kinase A (PKA), protein kinase C-α (PKCα), PKCδ, AKT, and p38 MAPK pathways 32. Inhibition of p38 MAPK, but not other A2BR downstream signaling pathways, phenocopied the effect of alloxazine in blocking paclitaxel-induced NANOG, SOX2, and KLF4 expression in both cells (Figure 5A and Figure S5A), suggesting that the regulatory role of A2BR on pluripotency factor expression may be through activation of p38 MAPK. Paclitaxel and adenosine, both of which activates A2BR, increased p38 MAPK phosphorylation in NTC but not in A2BR knockdown subclones of MDA-MB-231 cells (Figure 5B-C), confirming that p38 MAPK is activated in an A2BR-dependent manner. Inhibition of p38 MAPK by its specific inhibitor SB203580 abrogated paclitaxel-induced FOXO3 binding on *NANOG*, *SOX2*, and *KLF4* gene in both MDA-MB-231 (Figure 5D) and SUM159 (Figure S5B), which phenocopied the effect of A2BR knockdown (Figure 3B and Figure S2A).

To explore the role of p38 MAPK in A2BR-mediated pluripotency factor expression and BCSC enrichment in response to chemotherapy in vivo, we treated MMTV-PyMT transgenic mice with 5 mg/kg paclitaxel, alone or in combination with 10 mg/kg p38 MAPK specific inhibitor LY2228820. Although LY2228820 only slightly inhibited tumor growth rate (Figure 5E), it markedly inhibited paclitaxel-induced increases of ALDH^+^ (Figure 5F) and mammosphere-forming (Figure 5G) cells and of expression of Nanog, Sox2, and Klf4 (Figure 5H). Taken together, these data demonstrate that activation of p38 MAPK is involved in A2BR-mediated pluripotency factor expression and BCSC enrichment in response to chemotherapy.

### A2BR-mediated p38 MAPK activation promotes SMARCD3 nuclear translocation and FOXO3 recruitment to pluripotency factor genes

**Figure 6:**
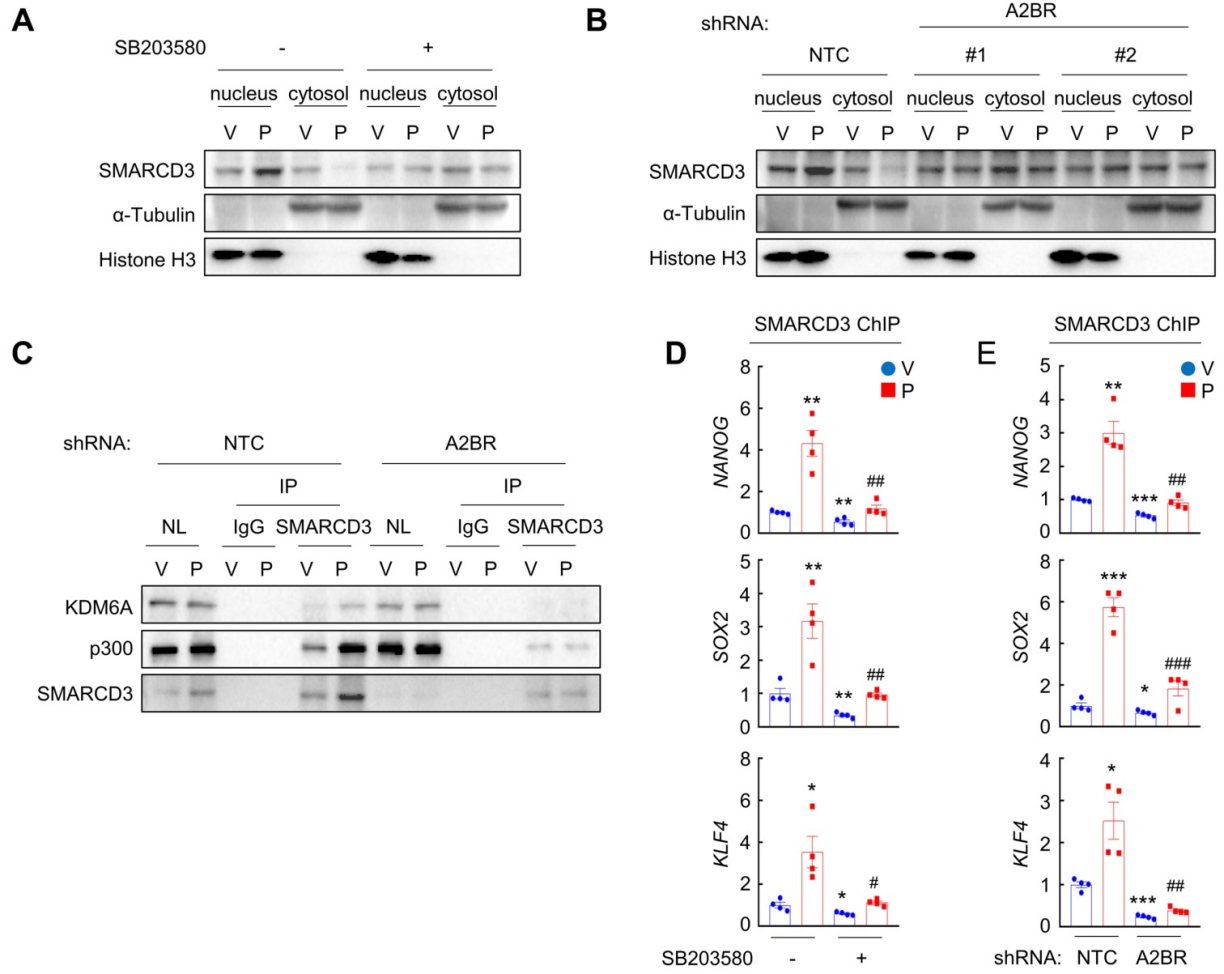
** Chemotherapy-induced A2BR expression and p38 activation promotes SMARCD3 nuclear translocation and binding on pluripotency factor genes. A,** MDA-MB-231 cells were treated with vehicle (V) or 10 nM paclitaxel (P), in the absence (-) or presence (+) of SB203580, for 72 h. Cytosolic and nuclear lysates were prepared, and immunoblot assays were performed to detect SMARCD3 subcellular localization. **B,** MDA-MB-231 NTC or A2BR knockdown subclones were treated with V or 10 nM P for 72 h. Cytosolic and nuclear lysates were prepared, and immunoblot assays were performed to detect SMARCD3 subcellular localization. **C,** MDA-MB-231 NTC or A2BR knockdown subclones were treated with V or 10 nM P for 72 h and nuclear lysates were prepared. Immunoprecipitation (IP) was performed using SMARCD3 antibody or control IgG followed by immunoblot assays. NL, nuclear protein lysate. D, MDA-MB-231 cells were treated with 10 nM P, 5 μM SB203580, or both for 72 h. ChIP was performed using SMARCD3 antibody followed by qPCR with primers flanking FOXO3 binding sites in the *NANOG*, *SOX2* and* KLF4* gene (mean ± SEM; n = 4); *p < 0.05, **p < 0.01 vs. V; ^#^p < 0.05, ^##^p < 0.01 vs. P. **E,** MDA-MB-231 NTC or A2BR knockdown subclones were treated with V or 10 nM P for 72 h. ChIP was performed using SMARCD3 antibody followed by qPCR with primers flanking FOXO3 binding sites in the *NANOG*, *SOX2* and* KLF4* gene (mean ± SEM; n = 4); *p < 0.05, **p < 0.01, ***p < 0.001 vs. NTC-V; ^#^p < 0.05, ^##^p < 0.01, ^###^p < 0.001 vs. NTC-P.

We next investigated how A2BR-mediated activation of p38 MAPK regulates epigenetic regulation of pluripotency factor genes. The chromatin remodeling factor SMARCD3 is a known substrate of p38 MAPK and translocates into nucleus, where it functions to regulate chromatin structure, upon phosphorylation by p38 MAPK 33. We found that in MDA-MB-231 cells, paclitaxel treatment induced SMARCD3 nuclear translocation, which was blocked by coadministration of p38 inhibitor SB203580 (Figure 6A). A2BR knockdown also abrogated paclitaxel-induced SMARCD3 nuclear translocation (Figure 6B), confirming that nuclear translocation of SMARCD3 is regulated by A2BR-p38 MAPK in response to chemotherapy in MDA-MB-231 cells.

Next, we studied the function of nuclear SMARCD3. We performed a co-IP assay in MDA-MB-231 nuclear lysates with an antibody against SMARCD3, and demonstrated that SMARCD3 interacted with KDM6A and p300 (Figure 6C). Paclitaxel treatment further increased SMARCD3 that interacted with KDM6A and p300, without altering KDM6A or p300 protein levels in the nucleus (Figure 6C). Knockdown of A2BR abrogated paclitaxel-induced SMARCD3 interaction with KDM6A and p300 (Figure 6C). ChIP-qPCR assays further showed that SMARCD3 protein occupied FOXO3 binding sites on the *NANOG*, *SOX2*, and *KLF4* genes, and paclitaxel treatment induced SMARCD3 binding to these sites in a p38 MAPK- and A2BR-dependent manner in MDA-MB-231 (Figure 6D-E) and SUM159 (Figure S6A-B) cells.

**Figure 7:**
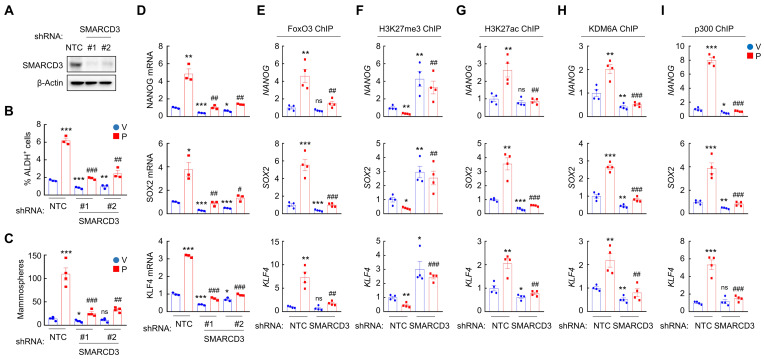
** SMARCD3 knockdown blocks paclitaxel-induced FOXO3 binding on pluripotency factor genes and inhibits BCSC enrichment. A,** MDA-MB-231 cells were transfected with vector encoding NTC or either of two shRNAs targeting SMARCD3 (#1 and #2), and immunoblot assay was performed. **B-D,** MDA-MB-231 NTC or SMARCD3 knockdown subclones were treated with vehicle (V) or 10 nM paclitaxel (P) for 72 h, and ALDH (B; mean ± SEM; n = 3), mammosphere (C; mean ± SEM; n = 4), and qPCR (D; mean ± SEM; n = 3) assays were performed; *p < 0.05, **p < 0.01, ***p < 0.001 vs. NTC-V; ^#^p < 0.05, ^##^p < 0.01, ^###^p < 0.001 vs. NTC-P; ns, not significant. **E-I,** MDA-MB-231 NTC or SMARCD3 knockdown subclones were treated with V or 10 nM P for 72 h. ChIP was performed using antibodies against FOXO3 (E), H3K27me3 (F), H3K27ac (G), KDM6A (H), or p300 (I), followed by qPCR with primers flanking FOXO3 binding sites in the *NANOG*, *SOX2* and* KLF4* gene (mean ± SEM; n = 4); *p < 0.05, **p < 0.01, ***p < 0.001 vs. NTC-V; ^##^p < 0.01, ^###^p < 0.001 vs. NTC-P; ns, not significant.

To determine the role of SMARCD3 in A2BR-mediated pluripotency factor expression and BCSC enrichment in response to chemotherapy, we generated two independent SMARCD3 knockdown subclones in MDA-MB-231 (Figure 7A) and SUM159 (Figure S7A) cells. SMARCD3 knockdown blocked paclitaxel-induced enrichment of ALDH^+^ (Figure 7B and Figure S7B) and mammosphere-forming (Figure 7C and Figure S7C) cells, and abrogated paclitaxel-induced NANOG, SOX2, and KLF4 mRNA expression (Figure 7D and Figure S7D). Mechanistically, SMARCD3 knockdown blocked paclitaxel-induced FOXO3 (Figure 7E and Figure S7E), KDM6A (Figure 7H and Figure S7H), and p300 (Figure 7I and Figure S7I) binding to *NANOG*, *SOX2*, and *KLF4* genes, and increased H3K27me3 (Figure 7F and Figure S7F) and decreased H3K27ac (Figure 7G and Figure S7G) marks on FOXO3 binding site of these genes. Taken together, these data demonstrate that chemotherapy induces SMARCD3 nuclear translocation and recruitment to FOXO3 binding sites of *NANOG*, *SOX2*, and *KLF4* genes in a A2BR- and p38 MAPK-dependent manner, leading to epigenetic regulation and transcriptional activation of these genes.

### A2BR is associated with poor clinical outcome in TNBC patients

**Figure 8:**
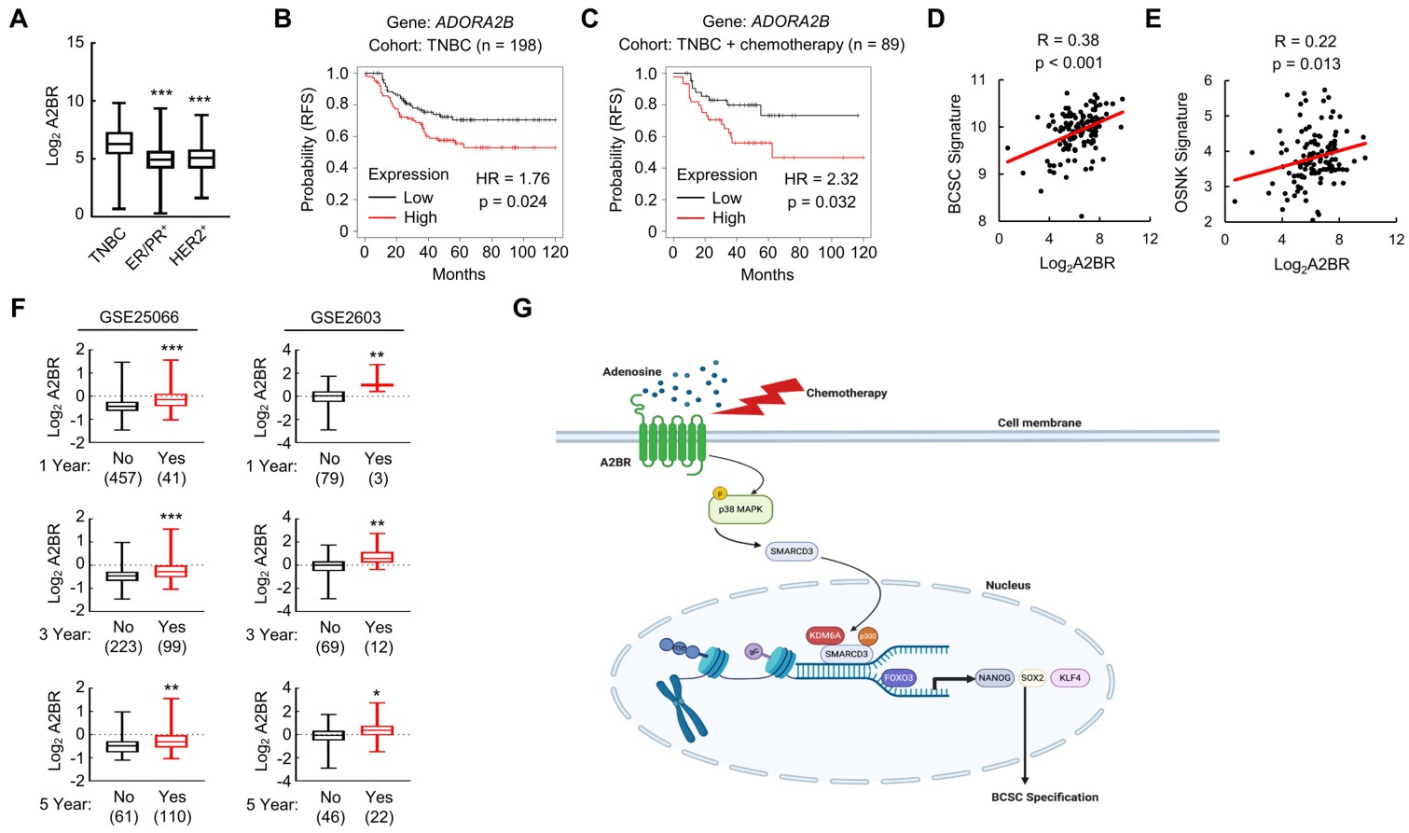
** A2BR is associated with poor clinical outcome in TNBC patients. A,** A2BR mRNA expression in different subtypes of breast cancer (TNBC, n= 123; ER/PR^+^, n = 615; HER2^+^, n = 124) from 1,215 human breast cancer specimens in the TCGA database was compared; ***p < 0.001 vs. TNBC. **B** and** C,** Kaplan-Meier analysis of relapse-free survival (RFS) was performed based on clinical and molecular data from 198 TNBC patients (B) or from a subgroup of 89 TNBC patients who received chemotherapy (C). The patients were stratified by A2BR mRNA levels in the primary tumor, which were greater (red) or less (black) than the median level. The hazard ratio (HR) and P value (log-rank test) are shown. **D** and** E,** Levels of A2BR mRNA, BCSC signature composed of transcripts of 20 genes, and OSNK signature (mRNA of pluripotency factor genes *NANOG*, *SOX2*, *OCT4*, and *KLF4*) were accessed from TCGA database. Correlation of A2BR mRNA with BCSC signature (D), and OSNK signature (E) were analyzed by Pearson's test. **F,** Clinical and molecular data from 2 datasets were accessed from GEO. A2BR mRNA levels in the primary breast cancer from patients who had metastasis (Yes) at year 1, 3, or 5 was compared with patients who had no metastasis (No) at the same time point; *p < 0.05, **p < 0.01, ***p < 0.001. **G,** A proposed model of A2BR in the epigenetic regulation of pluripotency factor gene expression and BCSC specification in response to chemotherapy.

We analyzed gene expression data from 1,247 primary human breast cancers in the Cancer Genome Atlas (TCGA) database and compared A2BR expression pattern in different subtypes of breast cancer. A2BR expression is significantly higher in TNBC compared with ER/PR^+^ and HER2^+^ breast cancer (Figure 8A), highlighting its important role in TNBC. To determine the clinical relevance of A2BR expression with regard to treatment outcomes in TNBC, we interrogated microarray data from 198 TNBC specimens and analyzed the correlation between A2BR expression and survival of TNBC patients 34. A2BR level above the median was significantly associated with decreased relapse-free survival in the cohort of TNBC patients (Figure 8B), with an even larger survival difference when only TNBC patients who received chemotherapy were analyzed (Figure 8C). To investigate the involvement of A2BR in the regulation of BCSCs in primary breast cancer, we analyzed the correlation of A2BR expression with a 20-gene BCSC signature 35, and with the OSNK signature that composed of the expression of pluripotency factors OCT4, SOX2, NANOG, and KLF4, from human TNBC samples in TCGA database, and found that A2BR expression was strongly correlated with the BCSC signature (Figure 8D) and the OSNK signature (Figure 8E). Because BCSCs play critical roles in the formation of clinically relevant metastasis, we also analyzed Gene Expression Omnibus (GEO) datasets 36,37 and found that breast cancer patients that developed metastasis within 1, 3 or 5 years had higher A2BR expression in their primary tumor compared with those who did not have metastasis at the same time point (Figure 8F). Taken together, these data demonstrate that A2BR expression is associated with the BCSC phenotype, tumor metastasis, and adverse clinical outcome in TNBC patients.

## Discussion

Although most TNBC patients respond to chemotherapy initially, a paradox in the treatment of TNBC is that the objective clinical responses that are majorly evaluated by tumor shrinkage often fail to translate into a substantial improvement of overall survival 38. This is due in part to the tumor heterogeneity and the existence of chemotherapy-resistant BCSCs 5,39, which are the source of cancer recurrence and metastasis. Chemotherapy induces an active conversion from non-stem cells to BCSCs, further potentiating cancer recurrence, metastasis, and patient mortality 40. Our previous studies have demonstrated transcriptional activation of genes encoding the pluripotency factors NANOG, SOX2, and KLF4 as the underlying mechanism of chemotherapy-induced BCSC enrichment 8-10. In the present study, we have delineated a molecular pathway that adenosine receptor A2BR promotes BCSC specification through epigenetic activation of pluripotency factor genes in TNBC. Chemotherapy treatment induces A2BR expression and activates its downstream p38 MAPK signaling pathway that mediates SMARCD3 nuclear translocation. Nuclear SMARCD3 recruits KDM6A and p300 to FOXO3-binding sites on *NANOG*, *SOX2*, and *KLF4* genes and decreases H3K27me3 and increases H3K27ac marks of these sites, leading to increased chromatin accessibility, increased FOXO3 binding, and transcriptional activation of these genes, and finally BCSC specification (Figure 8G). Our findings provide strong support for the therapeutic strategy of targeting A2BR in TNBC to improve the efficacy of chemotherapy through inhibition of chemotherapy-induced BCSC enrichment.

A2BR is a GPCR that coupled to G_s_ subtype of Gα protein 41. Upon binding with its ligand adenosine, the classical A2BR downstream signaling pathway is to increase production of cyclic AMP (cAMP) that activates PKA 42. However, inhibition of PKA, as well as several other protein kinases that have been reported to be activated downstream of A2BR, including PKCα, PKCδ, and AKT 32, failed to phenocopy A2BR inhibition in blocking paclitaxel-induced expression of pluripotency factors (Figure 5A and Figure S5A), suggesting that a novel effector rather than those common A2BR downstream effectors contributes to A2BR-induced pluripotency factor expression and the BCSC phenotype in response to chemotherapy. We demonstrated that pharmacological inhibition of p38 MAPK, which is activated by chemotherapy in an A2BR-dependent manner (Figure 5B), blocks paclitaxel-induced pluripotency factor expression and BCSC enrichment in vitro and in vivo (Figure 5D-H), suggesting that p38 MAPK is involved in transducing signal from A2BR to transcriptional activation of pluripotency factor genes. Although adenosine has been reported to activate p38 MAPK signaling through binding with A2BR 43,44, our present results suggest a different mechanism of A2BR-mediated p38 activation in response to chemotherapy that is independent of PKA or PKC. The molecular mechanism through which p38 MAPK is activated upon A2BR activation in response to chemotherapy, especially the type of Gα protein that coupled to A2BR, need to be delineated in the future study.

The MAPK signaling pathways are associated with aggressive phenotypes and chemoresistance in TNBC 45. The MAPK signaling pathway in mammalian cells includes three major branches: extracellular signal-regulated kinase (ERK), c-Jun N-terminal kinase (JNK), and p38 MAPK. Our previous study demonstrated that chemotherapy activates p38 MAPK but inhibits ERK signaling pathway in breast cancer 8,10. Although inhibition of ERK may decrease primary breast tumor growth, concern has been raised that it may have anti-therapeutic effect through enrichment of BCSC population, which increases the risk of cancer metastasis 8. Our current study provides strong evidence that pharmacological inhibition of p38 is effective in blocking chemotherapy-induced BCSC enrichment in an autochthonous breast cancer mouse model, suggesting p38 MAPK as a better potential target in combination with chemotherapy in TNBC. Further studies are warranted to evaluate the efficacy of different p38 inhibitors, including those that are currently in clinical trials 46, in the eradication of BCSCs in combination with chemotherapy in TNBC.

In eukaryotic cells, nucleosome is the structural unit of chromatin and is composed of 146 base pairs of duplex DNA wrapped around a histone octamer 47. Compaction of genomic DNA means that genes must become accessible for transcription machinery to be transcribed, and this process is controlled majorly by two ways: chromatin structure remodeling and histone modification 19. Our results demonstrated an A2BR-mediated crosstalk between these two ways in the regulation of pluripotency factor genes centered at one molecule SMARCD3. SMARCD3, also known as BAF60c, belongs to Brg1/Brm associated factors (BAFs) that are essential part of chromatin remodeling SWI/SNF complex 48. The BAF60 subunit is unique among all subunits of SWI/SNF complex and plays an important role as a linker between the SWI/SNF core complex and transcription factors to regulate target gene expression 49,50. SMARCD3/BAF60c has been reported to be a p38 MAPK substrate 33. Phosphorylation of SMARCD3 by p38 leads to its nuclear translocation, however, the biological consequence remains elusive. In the present study, we found a novel function of SMARCD3 as a scaffold protein in chromatin to recruit histone demethylase KDM6A and histone acetyltransferase p300, and to reciprocally regulate H3k27 tri-methylation and acetylation, leading to increased chromatin accessibility to transcription factor FOXO3.

Interestingly, recruitment of KDM6A and p300 does not happen globally but on specific genes (*NANOG*, *SOX2*, and *KLF4* genes in our present study), although nuclear SMARCD3 protein level increases in response to chemotherapy treatment. A caveat of this study is that the mechanism of the specificity has not been determined. It is important to elucidate SMARCD3 binding sites at genome-wide level and to determine genes that are regulated by SMARCD3 in response to chemotherapy treatment. A ChIP-sequencing experiment is warranted to determine whether SMARCD3 is recruited to specific genes or whether SMARCD3 is recruited to chromatin globally but only interact with histone modification enzymes at specific sites. In addition, our present study demonstrated the interaction of FOXO3 with KDM6A and p300 (Figure 4D). Whether KDM6A and p300 recruitment is required for FOXO3 transcriptional activity globally or only for specific genes is still elusive and is warranted to be investigated by ChIP-sequencing.

BCSCs must escape from anti-tumor immune surveillance to form a metastatic tumor, and adenosine-A2BR signaling is known as an important modulator of immune cell function 51. Chemotherapy induces adenosine production in TNBC cells through increased expression of CD39 and CD73 24, and adenosine treatment is sufficient to induce BCSC enrichment in an A2BR-dependent manner 27. Our current work is majorly focused on A2BR-mediated intrinsic mechanism, i.e., the role of A2BR expression in cancer cells but not in immune cells, for BCSC enrichment in response to chemotherapy. Although the role of A2BR expression in immune evasion is not directly evaluated, we performed in vivo studies with immunocompetent mouse model to evaluate the effects of A2BR inhibitor and p38 inhibitor in combination with chemotherapy, and the results are consistent with those from immunodeficient mice. Additional studies on A2BR expression in immune cells in response to chemotherapy will help us to fully understand the function of A2BR in chemotherapy-induced BCSC enrichment and metastasis.

In summary, we have delineated a signaling pathway downstream of A2BR that contributes to chemotherapy-induced BCSC enrichment through epigenetic regulation of pluripotency factor genes. A2BR is an attractive therapeutic target due to its cell membrane localization and its feasibility to be targeted. Our current study provides convincing evidence that genetic or pharmacological inhibition of A2BR effectively blocked chemotherapy-induced BCSC enrichment in both immunodeficient and immunocompetent mice. Clinical trials are warranted to evaluate the efficacy of pharmacological A2BR inhibitors, especially in combination with chemotherapy in TNBC. Taken together, our current study provides compelling evidence in support of the hypothesis that coadministration of A2BR inhibitor with chemotherapy may effectively inhibit BCSC enrichment and thereby increase the survival of women with TNBC.

## Methods and Materials

### Cell culture and reagents

MDA-MB-231 cells were maintained in DMEM; SUM159 and SUM149 cells were maintained in DMEM/F12 (50:50), both of which were supplemented with 10% fetal bovine serum and 1% penicillin-streptomycin. Cells were maintained at 37°C in a 5% CO_2_, 95% air incubator (20% O_2_). All chemicals are listed in Table S1.

### Lentiviral transduction

pLKO.1-puro lentiviral vectors encoding shRNA targeting A2BR and SMARCD3 were purchased from Sigma-Aldrich, and the clone IDs are shown in Table S2. Empty pLx304 vector was purchased from Addgene and pLx304 vector encoding A2BR was purchased from DNASU. Lentiviruses were packaged in 293T cells and viral supernatant was collected 48 h after transfection. MDA-MB-231 and SUM159 cells were transduced with viral supernatant supplemented with 8 μg/mL Polybrene (MilliporeSigma). After 24 h, cells were replenished with fresh medium containing 0.5 μg/mL puromycin (MilliporeSigma) and maintained in puromycin-containing medium for selection of stably transfected clones.

### Immunoblot assay

Cultured cells were lysed in RIPA buffer (EMD Millipore), whereas tumor tissues were lysed in RIPA buffer and homogenized with an electric homogenizer. Proteins (50 μg) were separated by SDS-PAGE, blotted onto nitrocellulose membranes, and probed with primary antibodies (Table S3). The membranes were then probed with horseradish peroxidase-conjugated secondary antibodies (GE Healthcare) and the chemiluminescent signal was detected using ECL plus (GE Healthcare).

### Reverse transcription and quantitative PCR

Total RNA was extracted with TRIzol (Invitrogen), precipitated with isopropanol, and treated with DNase (DNA-free, Invitrogen). cDNA was synthesized with cDNA reverse transcription kit (Applied Biosystems) from 1 μg total RNA and qPCR analysis was performed using SYBR Green and the CFX96 Touch real-time PCR detection system (Bio-Rad). The expression of each target mRNA relative to 18S rRNA was calculated based on the cycle threshold (C_t_) as 2^-Δ(ΔC^_t_^)^, in which ΔC_t_ = C_t_ (target) - C_t_ (18S rRNA), and Δ(ΔC_t_) = ΔC_t_ (test sample) - ΔC_t_ (control sample). Primer sequences (5' to 3') for RT-qPCR are shown in Table S4.

### Nuclear and cytoplasmic fractionation

Cultured cells were resuspended in hypotonic buffer (10 mM HEPES, 1.5 mM MgCl_2_, 10 mM KCl, 0.5 mM DTT, 0.05% NP40, pH 7.9) with protease inhibitor mixture (Roche) on ice for 10 min and centrifuged at 4°C at 3000 rpm for 10 min. The supernatants were saved as cytoplasmic fraction. Pellets were resuspended in high salt cell extraction buffer (5 mM HEPES, 1.5 mM MgCl_2_, 0.2 mM EDTA, 0.5 mM DTT, 26% glycerol, 300 mM NaCl, pH 7.9) with protease inhibitor mixture, homogenized with 30 full strokes in Dounce homogenizer, incubated on ice for 30 min, and centrifuged at 15,000 rpm for 30 min at 4°C. Supernatants were saved as nuclear fraction.

### Co-IP

Equal amounts of nuclear protein lysates (500 μg) were incubated with control IgG or antibodies against FOXO3 (Novus, NBP2-16521) or SMARCD3 (Santa Cruz, sc-101163) in the presence of protein G-Sepharose beads (Amersham Biosciences) at 4 °C overnight, and the resulting immunoprecipitates were then subjected to immunoblot assays.

### Aldehyde dehydrogenase (ALDH) assay

The ALDH assay was performed according to manufacturer's instructions (Aldefluor, Stem Cell Technologies). Cultured cells were trypsinized, whereas tumor tissues were minced, digested with 1 mg/mL of type 1 collagenase (Sigma-Aldrich) at 37°C for 30 min, and filtered through a 70-μm cell strainer. 5 x 10^5^ cells were suspended in assay buffer containing 0.5 μM BODIPY-animoacetaldehyde and incubated at 37°C for 45 minutes. An aliquot of cells from each sample was treated with 50 mM diethylaminobenzaldehyde, an ALDH inhibitor, as a negative control for gating. Samples were analyzed by flow cytometry using FACScalibur (BD Biosciences).

### Mammosphere assay

Cultured cells were trypsinized, whereas tumor tissues were minced, digested with 1 mg/mL of type 1 collagenase (Sigma-Aldrich) at 37 °C for 30 min, and filtered through a 70-μm cell strainer. The number of live cells was determined using trypan blue staining and single cell suspensions were seeded in six-well ultra-low-attachment plates (Corning) at a density of 5,000 cells/mL in complete MammoCult Medium (Stem Cell Technologies). Mammosphere cultures were photographed 7 days later using a phase contrast microscope (Olympus) and mammospheres ≥ 50 μm in diameter were counted using ImageJ software (NIH).

### Chromatin immunoprecipitation (ChIP) assay

Cultured cells or minced tumor tissues were cross-linked in 3.7% formaldehyde for 15 minutes, quenched in 0.125 mol/L glycine for 5 minutes and lysed with SDS lysis buffer. Chromatin was sheared by sonication, sonicated lysates were precleared with salmon sperm DNA/protein A agarose slurry (EMD Millipore) for 1 hour and incubated with antibodies (Table S5) in the presence of agarose beads overnight. After sequential washing of the agarose beads, DNA was eluted in 1% SDS/0.1 mol/L NaHCO3, and crosslinks were reversed by the addition of 0.2 mol/L NaCl. DNA was purified by phenol-chloroform extraction and ethanol precipitation, and candidate binding sites were analyzed by qPCR. Primer sequences (5' to 3') are shown in Table S6.

### Animal study

Animal protocols were approved by Institutional Animal Care and Use Committee of West China hospital, Sichuan University and were in accordance with the National Institute of Health Guide for the Care and Use of Laboratory Animals. For assays with SCID mice, 2 × 10^6^ MDA-MB-231 parental or knockdown subclone cells were injected into the MFP of 5-to-7 week-old female mice in a 1:1 suspension of Matrigel (BD Biosciences) in PBS. Mice were treated with drugs as indicated. For assays with MMTV-PyMT transgenic mice, mice were treated when the accumulated volume of mammary tumors in each mouse reached 150 mm^3^. Primary tumors were measured for length (L) and width (W), and tumor volume (V) was calculated as V = L × W^2^ × 0.524. Paclitaxel, carboplatin, and alloxazine were given by intraperitoneal injection; LY2228820 was given by oral gavage.

### Database analysis

Expression data for A2BR, NANOG, SOX2, OCT4, KLF4, and BCSC signature-gene mRNAs in different subtypes of primary breast cancers were accessed from The Cancer Genome Atlas (TCGA) invasive carcinoma gene expression dataset (cancergenome.nih.gov). Pearson test was performed to analyze the correlation of A2BR expression with the BCSC signature and OSNK signature in 123 TNBC patients. Kaplan-Meier curves were generated from a dataset containing gene expression and survival data from 3,951 breast cancer patients 33 and the log-rank test was performed. Expression of A2BR in primary breast cancer patient dataset GSE25066 35 and GSE2603 36 was accessed from the Gene Expression Omnibus (ncbi.nlm.nih.gov/geo/).

### Statistical analysis

All data are expressed as mean ± SEM. Differences between 2 groups were analyzed by 2-tailed Student's *t* test, whereas differences between multiple groups were analyzed by ANOVA with post hoc test. For all analyses, values of p < 0.05 were considered significant.

## Supplementary Material

Supplementary figures and tables.
