# Supplementary material for: Chemotherapy-induced adenosine A2B receptor expression mediates epigenetic regulation of pluripotency factors and promotes breast cancer stemness
Source: Theranostics. 2022 Feb 28;12(6):2598–612. doi: 10.7150/thno.70581 (PMC8965495; doi:10.7150/thno.70581)
Supplement: Supplementary file 1 — Supplementary figures and tables. [file thnov12p2598s1.pdf]

Supplementary information for

Chemotherapy-induced adenosine A2B receptor expression mediates epigenetic regulation of pluripotency factors and promotes breast cancer stemness

Jie Lan, Guangyao Wei, Jia Liu, Fan Yang, Rong Sun, Haiquan Lu

Corresponding Author: Haiquan Lu

Email: lvhaiquan@sdu.edu.cn

This file includes:

Supplementary figures S1-S7;

Supplementary figure legends S1-S7;

Supplementary tables S1-S6.

Figure S1

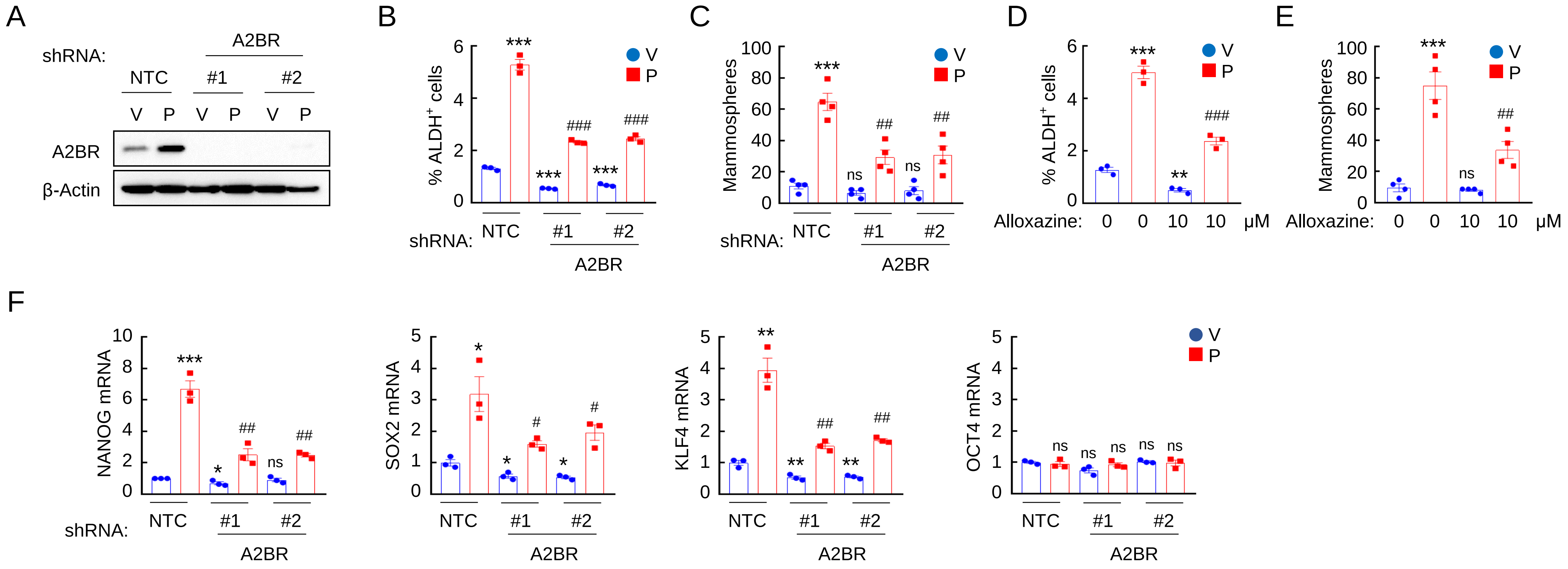

A
shRNA:
A2BR
NTC
#1
#2
V P V P V P
A2BR
β-Actin
B
% ALDH+ cells
V
P
***
###
###
***
***
shRNA:
NTC
#1
#2
A2BR
C
Mammospheres
***
ns
##
ns
##
D
% ALDH+ cells
***
**
###
Alloxazine: 0 0 10 10 μM
E
Mammospheres
***
ns
##
F
NANOG mRNA
SOX2 mRNA
KLF4 mRNA
OCT4 mRNA

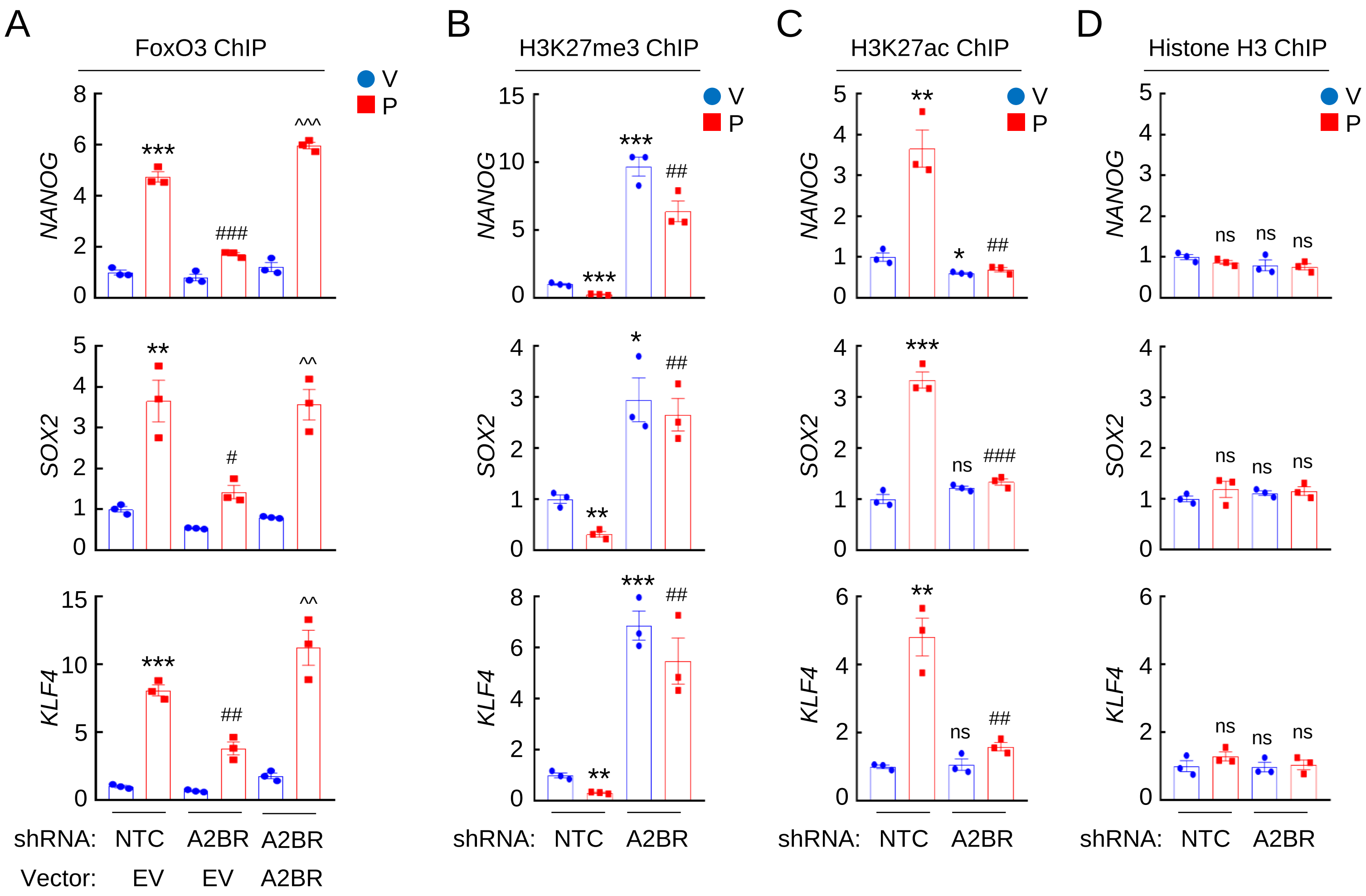
A
FoxO3 ChIP
B
H3K27me3 ChIP
C
H3K27ac ChIP
D
Histone H3 ChIP
V
P
NANOG
SOX2
KLF4
***
###
^^^
**
#
^^
##
*
ns
shRNA: NTC A2BR A2BR
Vector: EV EV A2BR
shRNA: NTC A2BR

Figure S3

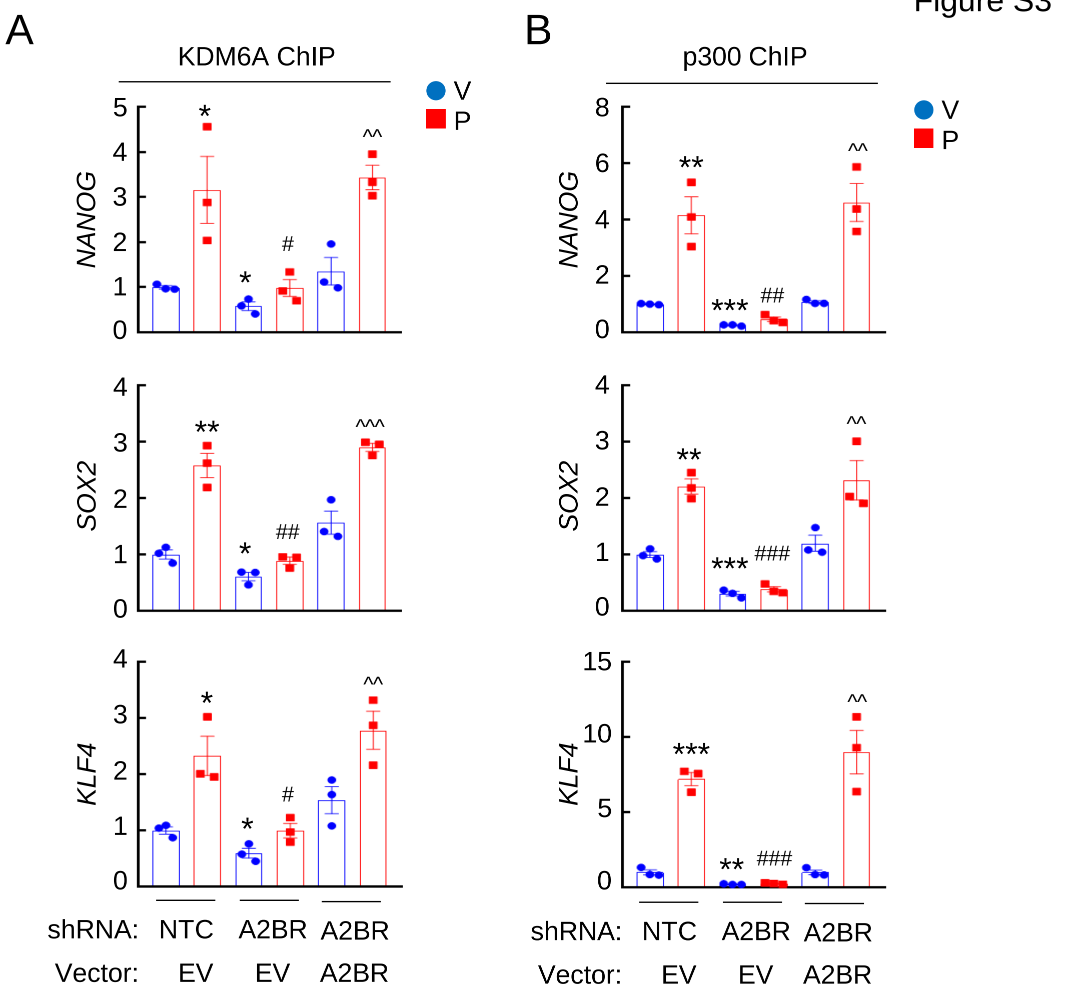
A
KDM6A ChIP
B
p300 ChIP
V
P
NANOG
SOX2
KLF4
shRNA: NTC A2BR A2BR
Vector: EV EV A2BR

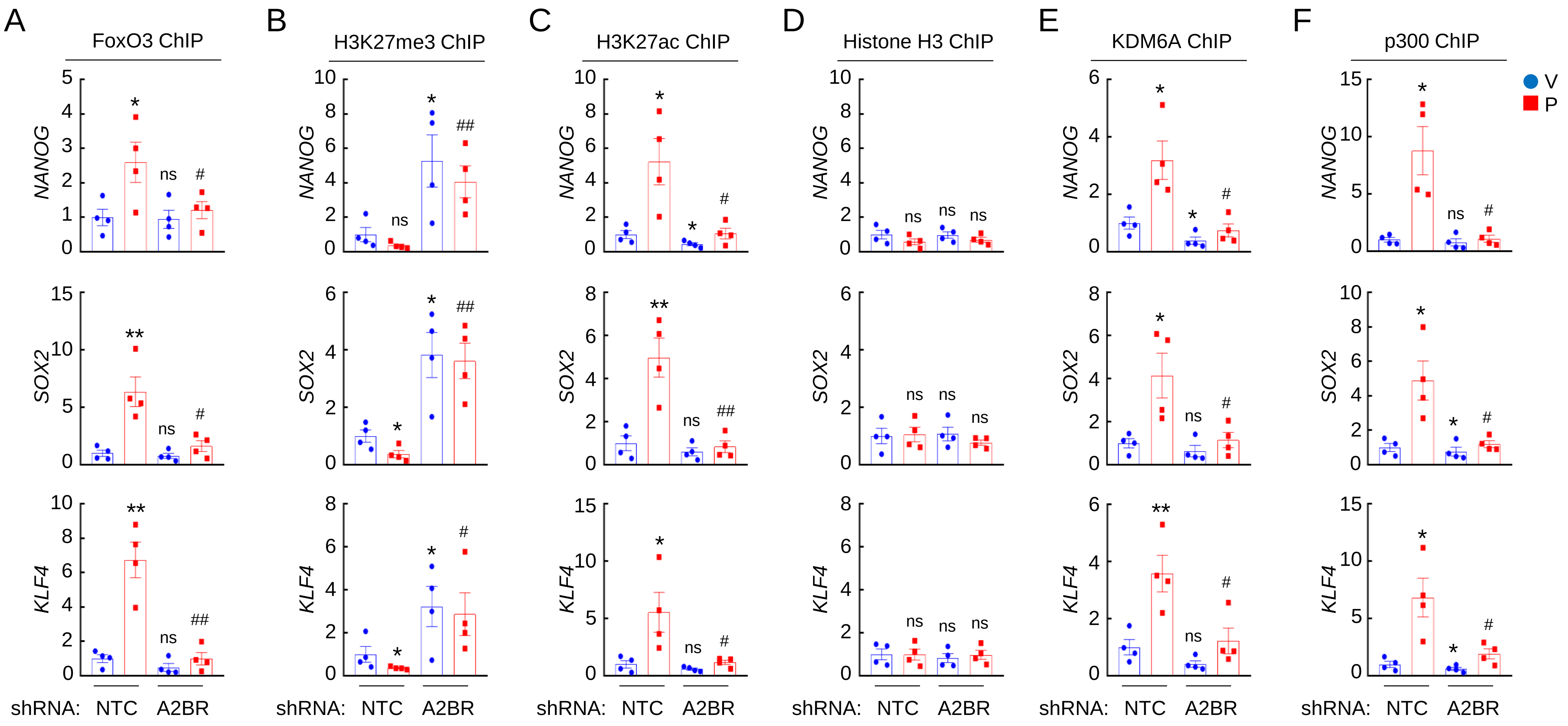
A
FoxO3 ChIP
B
H3K27me3 ChIP
C
H3K27ac ChIP
D
Histone H3 ChIP
E
KDM6A ChIP
F
p300 ChIP
V
P
NANOG
SOX2
KLF4
shRNA: NTC A2BR

Figure S5

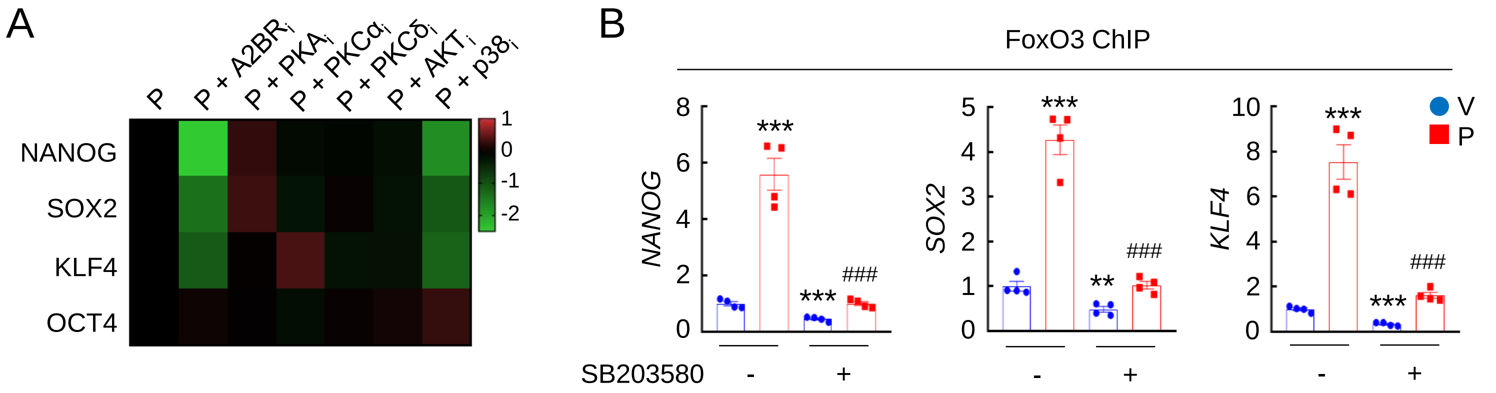
A
P
P + A2BRi
P + PKAi
P + PKCαi
P + PKCδi
P + AKTi
P + p38i
NANOG
SOX2
KLF4
OCT4
1
0
-1
-2
B
FoxO3 ChIP
NANOG
SOX2
KLF4
V
P
***
**
###
SB203580
-
+

Figure S6

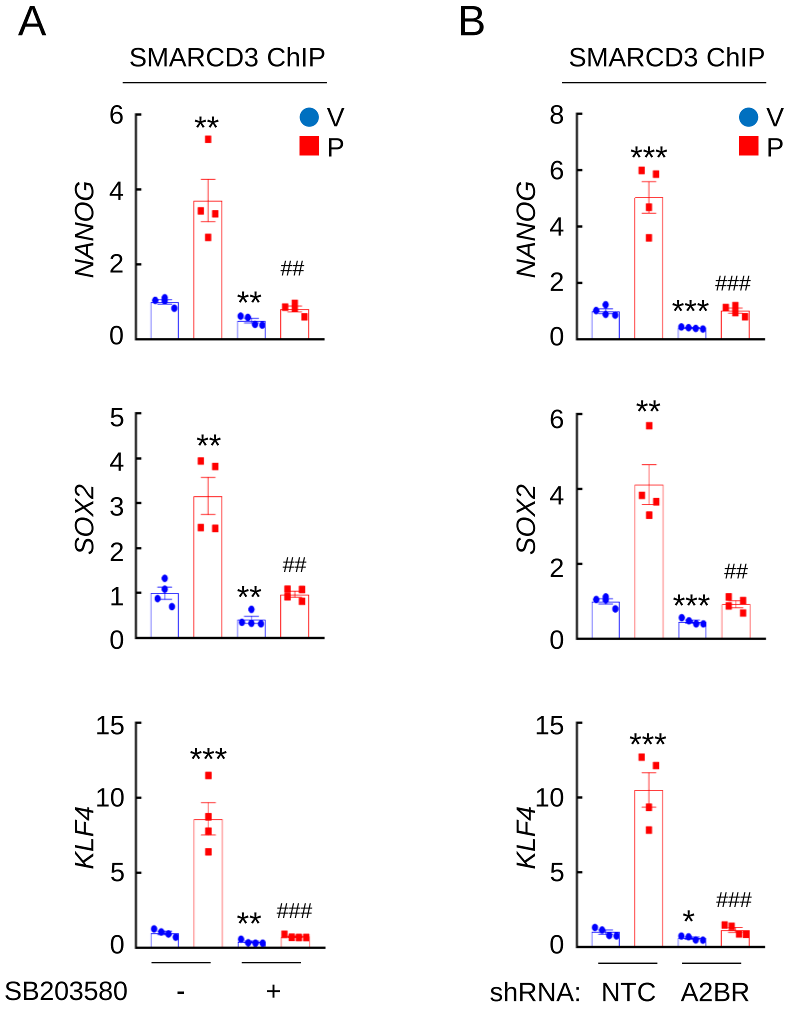

A
SMARCD3 ChIP
V
P
NANOG
SOX2
KLF4
**
***
##
###
SB203580
-
+
B
SMARCD3 ChIP
V
P
NANOG
SOX2
KLF4
*
**
***
##
###
shRNA:
NTC
A2BR

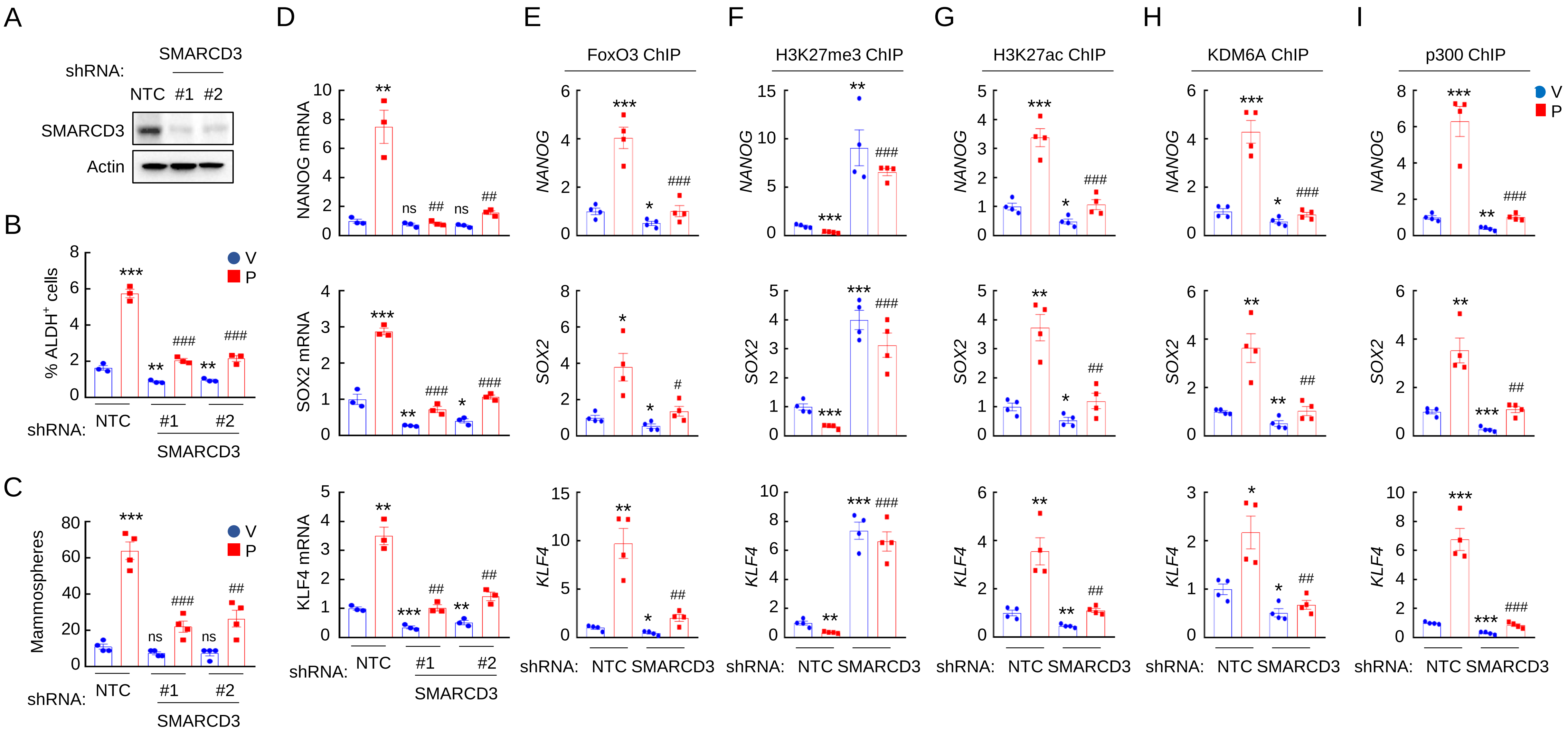
A
shRNA:
SMARCD3
NTC #1 #2
SMARCD3
Actin
B
% ALDH+ cells
V
P
shRNA: NTC #1 #2
SMARCD3
C
Mammospheres
V
P
shRNA: NTC #1 #2
SMARCD3
D
NANOG mRNA
SOX2 mRNA
KLF4 mRNA
shRNA: NTC #1 #2
SMARCD3
E
FoxO3 ChIP
F
H3K27me3 ChIP
G
H3K27ac ChIP
H
KDM6A ChIP
I
p300 ChIP
NANOG
SOX2
KLF4
shRNA: NTC SMARCD3

Supplementary Figure Legends

Figure S1. Chemotherapy-induced A2BR expression promotes pluripotency factor expression and the BCSC phenotype. A-C, SUM159 subclones stably transfected with vector encoding non-targeting control shRNA (NTC) or either of two different shRNAs targeting A2BR (#1 and #2) were treated with vehicle (V) or 5 nM paclitaxel (P) for 72 hours. Expression of A2BR protein (A), the percentage of $ALDH^+$ cells (B; mean ± SEM; n = 3) and the number of mammospheres formed per 1,000 cells seeded (C; mean ± SEM; n = 4) were determined; $^{***}p < 0.001$ vs. NTC-V; $^{\#\#}p < 0.01$, $^{\#\#\#}p < 0.001$ vs. NTC-P; ns, not significant. **D** and **E,** SUM159 cells were treated with 5 nM paclitaxel, 10 μM alloxazine, or both for 72 hours. The percentage of $ALDH^+$ cells (D; mean ± SEM; n = 3) and the number of mammospheres formed per 1,000 cells seeded (E; mean ± SEM; n = 4) were determined; $^{***}p < 0.001$ vs. V; $^{\#\#}p < 0.01$, $^{\#\#\#}p < 0.001$ vs. P; ns, not significant. **F,** SUM159 NTC or A2BR knockdown subclones were treated with V or 5 nM P for 72 hours, and RT-qPCR assays were performed (mean ± SEM; n = 3); $^{*}p < 0.05$, $^{**}p < 0.01$, $^{***}p < 0.001$ vs. NTC-V; $^{\#}p < 0.05$, $^{\#\#}p < 0.01$ vs. NTC-P; ns, not significant.

Figure S2. Chemotherapy-induced A2BR expression promotes FOXO3 binding on pluripotency factor genes through decreased H3K27me3 and increased H3K27ac chromatin marks. A, SUM159 NTC or A2BR knockdown subclones were transfected with pLX304 (empty vector, EV) or pLX304 encoding A2BR. Cells were treated with vehicle (V) or 5 nM paclitaxel (P) for 72 hours and chromatin immunoprecipitation (ChIP) was performed with FOXO3 antibody followed by qPCR with primers flanking

FOXO3 binding sites in the *NANOG*, *SOX2* and *KLF4* gene (mean ± SEM; n = 3); **p < 0.01, ***p < 0.001 vs. NTC-V; $^{\#}$p < 0.05, $^{\#\#}$p < 0.01, $^{\#\#\#}$p < 0.001 vs. NTC-P; $^{\wedge\wedge}$p < 0.01, $^{\wedge\wedge\wedge}$p < 0.0001 vs. A2BR shRNA/EV-P; ns, not significant. **B-D,** SUM159 NTC or A2BR knockdown subclones were treated with V or 5 nM P for 72 hours. ChIP was performed using antibodies against H3K27me3 (B), H3K27ac (C), or Histone H3 (D) followed by qPCR with primers flanking FOXO3 binding sites in the *NANOG*, *SOX2* and *KLF4* gene (mean ± SEM; n = 3); **p < 0.01, ***p < 0.001 vs. NTC-V; $^{\#\#}$p < 0.01, $^{\#\#\#}$p < 0.001 vs. NTC-P; ns, not significant.

Figure S3. A2BR regulates recruitment of KDM6A and p300 at FOXO3 binding sites of pluripotency factor genes. A and **B,** SUM159 NTC or A2BR knockdown subclones were transfected with pLX304 (empty vector, EV) or pLX304 encoding A2BR. Cells were treated with vehicle (V) or 5 nM paclitaxel (P) for 72 hours. ChIP was performed using antibodies against KDM6A (A) or p300 (B) followed by qPCR with primers flanking FOXO3 binding sites in the *NANOG*, *SOX2* and *KLF4* gene (mean ± SEM; n = 3); *p < 0.05, **p < 0.01, ***p < 0.001 vs. NTC-V; $^{\#}$p < 0.05, $^{\#\#}$p < 0.01, $^{\#\#\#}$p < 0.001 vs. NTC-P; $^{\wedge\wedge}$p < 0.01, $^{\wedge\wedge\wedge}$p < 0.0001 vs. A2BR shRNA/EV-P; ns, not significant.

Figure S4. A2BR decreases H3K27me3 and increases H3K27ac marks and promotes FOXO3 binding on pluripotency factor genes in vivo. A-F, 2×10^6 MDA-MB-231 NTC or A2BR knockdown subclone cells were implanted into the MFP of SCID mice. When tumor volume reached 200 mm^3 (day 0), mice were grouped randomly and treated with vehicle (V) or paclitaxel (P; 10 mg/kg, days 0, 5, and 10). Tumors were harvested on day 13, and ChIP was performed using antibodies against FOXO3 (A),

H3K27me3 (B), H3K27ac (C), Histone H3 (D), KDM6A (E), or p300 (F) followed by qPCR with primers flanking FOXO3 binding sites in the *NANOG*, *SOX2* and *KLF4* gene (mean ± SEM; n = 4); $^{*}p < 0.05$, $^{**}p < 0.01$ vs. NTC-V; $^{\#}p < 0.05$, $^{\#\#}p < 0.01$ vs. NTC-P; ns, not significant.

Figure S5. Inhibition of p38 MAPK blocks paclitaxel-induced FOXO3 binding and expression of pluripotency factor genes. A, SUM159 cells were treated with 5 nM paclitaxel (P), alone or in combination with 10 μM alloxazine ($A2BR_i$), 2.5 μM H-89 (PKA_i), 1 μM Gö6983 ($PKC\alpha_i$), 1 μM rottlerin ($PKC\delta_i$), 1 μM MK-2206 (AKT_i), or 5 μM SB203580 ($p38_i$), for 72 hours. RT-qPCR assays were performed and $\log_2$ fold change of mRNA expression of pluripotency factors (vs. P) is presented as a heat map. **B,** SUM159 cells were treated with 5 nM paclitaxel, 5 μM SB203580, or both for 72 hours. ChIP was performed using FOXO3 antibody followed by qPCR with primers flanking FOXO3 binding sites in the *NANOG*, *SOX2* and *KLF4* gene (mean ± SEM; n = 4); $^{**}p < 0.01$, $^{***}p < 0.001$ vs. V; $^{\#\#\#}p < 0.001$ vs. P.

Figure S6. p38 MAPK inhibition or A2BR knockdown blocks paclitaxel-induced SMARCD3 binding on pluripotency factor genes. A, SUM159 cells were treated with 5 nM paclitaxel (P), 5 μM SB203580, or both for 72 hours. ChIP was performed using SMARCD3 antibody followed by qPCR with primers flanking FOXO3 binding sites in the *NANOG*, *SOX2* and *KLF4* gene (mean ± SEM; n = 4); $^{**}p < 0.01$, $^{***}p < 0.001$ vs. V; $^{\#\#}p < 0.01$, $^{\#\#\#}p < 0.001$ vs. P. **B,** SUM159 NTC or A2BR knockdown subclones were treated with V or 5 nM P for 72 hours. ChIP was performed using SMARCD3 antibody followed by qPCR with primers flanking FOXO3 binding sites in

the *NANOG*, *SOX2* and *KLF4* gene (mean ± SEM; n = 4); *p < 0.05, **p < 0.01, ***p < 0.001 vs. NTC-V; ##p < 0.01, ###p < 0.001 vs. NTC-P.

Figure S7. SMARCD3 knockdown blocks paclitaxel-induced FOXO3 binding on pluripotency factor genes and inhibits BCSC enrichment. A, SUM159 cells were transfected with vector encoding NTC or either of two shRNAs targeting SMARCD3 (#1 and #2), and immunoblot assay was performed. **B-D,** SUM159 NTC or SMARCD3 knockdown subclones were treated with vehicle (V) or 5 nM paclitaxel (P) for 72 hours, and ALDH (B; mean ± SEM; n = 3), mammosphere (C; mean ± SEM; n = 4), and qPCR (D; mean ± SEM; n = 3) assays were performed; *p < 0.05, **p < 0.01, ***p < 0.001 vs. NTC-V; ##p < 0.01, ###p < 0.001 vs. NTC-P; ns, not significant. **E-I,** SUM159 NTC or SMARCD3 knockdown subclones were treated with V or 5 nM P for 72 hours. ChIP was performed using antibodies against FOXO3 (E), H3K27me3 (F), H3K27ac (G), KDM6A (H), or p300 (I), followed by qPCR with primers flanking FOXO3 binding sites in the *NANOG*, *SOX2* and *KLF4* gene (mean ± SEM; n = 4); *p < 0.05, **p < 0.01, ***p < 0.001 vs. NTC-V; ##p < 0.01, ###p < 0.001 vs. NTC-P; ns, not significant.

Table S1. Chemical information.

Chemical	Manufacture	Catalog #
Paclitaxel	MilliporeSigma	T7402
Carboplatin	MilliporeSigma	C2538
Alloxazine	MilliporeSigma	A28651
H-89	Cayman Chemical	S1582
Gö6983	Santa Cruz	sc-203432
rottlerin	SelleckChem	S7862
MK-2206	SelleckChem	S1078
SB203580	Enzo	BML-EI286
LY2228820	APExBio	A5566

Table S2. Oligonucleotide sequence of shRNAs.

shRNA	Clone ID
ADORA2B #1	NM_000676.2-926s21c1
ADORA2B #2	NM_000676.2-1478s21c1
SMARCD3 #1	NM_003078.3-1190s21c1
SMARCD3 #2	NM_003078.3-1052s21c1

Table S3. Primary antibody information for immunoblot assays.

Antibody	Manufacture	Catalog #
A2BR	Novus Biologicals	NBP1-68953
NANOG	Novus Biologicals	AF1997
SOX2	Bioss	bs-0523R
KLF4	Novus Biologicals	NBP1-83940
OCT4	Novus Biologicals	NB100-2379
p-FoxO3 (S294)	Cell Signaling Technology	5538
FoxO3	Novus Biologicals	NBP2-16521
H3K27me3	Novus Biologicals	NBP2-16840
H3K27ac	Novus Biologicals	NBP2-54615
KDM6A	Novus Biologicals	NBP1-80628
p300	Novus Biologicals	NB100-616
p-p38 (Y182)	Novus Biologicals	NB100-82097
p38	Novus Biologicals	AF8691
SMARCD3	Santa Cruz	sc-101163
α-tubulin	Novus Biologicals	NB100-690
Histone H3	Novus Biologicals	NB500-171
Actin	Santa Cruz	sc-1616

Table S4. Oligonucleotide sequence of RT-qPCR primers.

Gene	sequence (5' to 3')
NANOG (human)	Forward: TTTGTGGGCCTGAAGAAAACT
	Reverse: AGGGCTGTCCTGAATAAGCAG
SOX2 (human)	Forward: GCCGAGTGGAAACTTTTGTCG
	Reverse: GGCAGCGTGTACTTATCCTTCT
KLF4 (human)	Forward: CGGACATCAACGACGTGAG
	Reverse: GACGCCTTCAGCACGAACT
OCT4 (human)	Forward: TTCAGCCAAACGACCATCTG
	Reverse: CACGAGGGTTTCTGCTTTGC
Nanog (mouse)	Forward: TCTTCCTGGTCCCCACAGTTT
	Reverse: GCAAGAATAGTTCTCGGGATGAA
Sox2 (mouse)	Forward: GCGGAGTGGAAACTTTTGTCC
	Reverse: CGGGAAGCGTGTACTTATCCTT
Klf4 (mouse)	Forward: GTGCCCCGACTAACCGTTG
	Reverse: GTCGTTGAACTCCTCGGTCT
Oct4 (mouse)	Forward: CGGAAGAGAAAGCGAACTAGC
	Reverse: ATTGGCGATGTGAGTGATCTG
18S	Forward: CGGCGACGACCCATTCGAAC
	Reverse: GAATCGAACCCTGATTCCCCGTC

Table S5. Primary antibody information for ChIP assays.

Antibody	Manufacture	Catalog #
FoxO3	Novus Biologicals	NBP2-16521
H3K27me3	Novus Biologicals	NBP2-16840
H3K27ac	Novus Biologicals	NBP2-54615
Histone H3	Novus Biologicals	NB500-171
KDM6A	Novus Biologicals	NBP1-80628
p300	Novus Biologicals	NB100-616
SMARCD3	Santa Cruz	sc-101163

Table S6. Oligonucleotide sequence of ChIP primers.

NANOG	Forward: TGAGGCAAAGAGCATTGTTG
	Reverse: TGATGAAGGAATGGGCATAA
SOX2	Forward: GCAATGCTCTTTTAAGGGAAA
	Reverse: GACTTCCGGAATGACCAAAG
KLF4	Forward: CCCAGTGTCCCCTCTCTTCT
	Reverse: CGTGAATGTGGTGTTCGGTA
